# Refined chronologies of magnetochron M0r reveal asynchronous terrestrial and marine carbon isotope responses to Oceanic Anoxic Event 1a

**DOI:** 10.1126/sciadv.aea8374

**Published:** 2026-03-04

**Authors:** Gui-Mei Lu, Zhong-Shan Shen, Cheng-Long Deng, James G. Ogg, Ming-Dao Sun, Ming-Song Li, Hua-Feng Qin, Ri-Xiang Zhu, Zhong-He Zhou, Yi-Gang Xu

**Affiliations:** ^1^ State Key Laboratory of Deep Earth Processes and Resources, Guangzhou Institute of Geochemistry, Chinese Academy of Sciences, Guangzhou 510640, China.; ^2^ State Key Laboratory of Lithospheric and Environmental Coevolution, Institute of Geology and Geophysics, Chinese Academy of Sciences, Beijing 100029, China.; ^3^ International Paleogeography Center, Institute of Sedimentary Geology, and Key Laboratory of Deep-time Geography and Environment Reconstruction, Chengdu University of Technology, Chengdu 610059, China.; ^4^ Department of Earth, Atmospheric, and Planetary Sciences, Purdue University, 550 Stadium Mall Drive, West Lafayette, West Lafayette, IN 47907-2051, USA.; ^5^ Key Laboratory of Orogenic Belts and Crustal Evolution, MOE, School of Earth and Space Sciences, Peking University, Beijing 100871, China.; ^6^ Key Laboratory of Vertebrate Evolution and Human Origins, Institute of Vertebrate Paleontology and Paleoanthropology, Chinese Academy of Sciences, Beijing 100044, China.

## Abstract

The base of magnetochron M0r has been informally used to calibrate the onset of Oceanic Anoxic Event 1a (OAE1a) and place the Barremian-Aptian boundary; however, its precise age remains a subject of debate. Here, we present a 1497.5-m continuous drill core from the Lower Cretaceous lacustrine Jiufotang Formation in North China. High-resolution cycle-calibrated magnetostratigraphic analyses precisely constrain the termination of Chron M0r to 121.26 ± 0.38 Ma. The carbon isotope excursion associated with OAE1a in the same core postdates the termination of Chron M0r by 1.24 ± 0.4 Myr, substantially longer than ~0.3 to 0.66 Myr offset observed in marine records. This refined chronology provides direct evidence for asynchronous carbon cycles between terrestrial and marine systems during OAE1a, thereby challenging the prevailing hypothesis of rapid atmospheric CO_2_ emissions at the onset of OAE1a.

## Teaser

Revised magnetochron M0r age reveals a decoupled carbon isotope response to OAE1a between terrestrial and marine systems.

## INTRODUCTION

The Aptian Stage is a critical interval in the middle Cretaceous, characterized by tremendous environmental and biotic perturbations (*1*). Notably, the early Aptian witnessed global Oceanic Anoxic Event 1a (OAE1a) (~120 million years ago), typified by the deposition of organic-rich black shales informally known as the Selli level, corresponding to the C3-C6 carbon isotope anomaly intervals observed in the Tethyan and Pacific realms (*2*–*6*). The onset of OAE1a is marked by a pronounced negative carbon isotope excursion (NCIE) recorded in marine carbonates (δ^13^C_carb_) and organic matter (δ^13^C_org_), designated as the C3 segment in carbon isotope stratigraphy (*2*, *7*–*9*). This excursion is commonly attributed to the influx of ^13^C-depleted carbon into the ocean-atmosphere system, in the aftermath of emplacement of the Ontong Java Plateau (OJP) large igneous province (LIP) (*8*, *10*–*12*). However, this causal relationship remains debated due to uncertainties in the precise timing of both OJP emplacement and the onset of OAE1a (*13*–*16*). In the marine realm, the onset of OAE1a has been astronomically tuned to the base (121 Ma) of the geomagnetic reversed-polarity Chron M0r (*8*, *15*). That base had been informally used for placing the Barremian-Aptian boundary before a revised ammonite biostratigraphy suggested that a better global correlation marker and boundary assignment could be the carbon-isotope excursion at the onset of OAE1a (*17*, *18*). The end of this Chron M0r denotes the start of the Cretaceous Normal Superchron (CNS), a unique period of no notable geomagnetic reversals that lasted ~38 Myr (*19*).

Despite substantial efforts by the geoscience community to determine the onset and termination ages of Chron M0r in marine and terrestrial records (*20*–*22*), its timeframe has long been highly contentious. For example, its onset age had ranged from around 119 Ma in the geologic timescale (GTS) 1983 (*23*) to 126.3 Ma in the GTS 2012 (*24*), before being placed at 121.40 Ma in the widely used GTS 2020 (*25*). The GTS 2020 assignment was partly based on the magnetostratigraphy in combination with ID-TIMS U-Pb dating of boreholes in Svalbard, Norway, which had estimated the onset of Chron M0r at 121.2 ± 0.4 Ma (*21*). However, later U-Pb and ^40^Ar/^39^Ar analyses of tuffs recording the M1n-M0r geomagnetic reversal in the Qingshan Group, Jiaolai Basin, eastern China (*26*), suggested an onset age of 120.29 ± 0.09 Ma for Chron M0r (*22*), which is ~1 Myr younger than that of the Svalbard study (*21*). The age discrepancies in dating Chron M0r introduce large uncertainties in reconstructing plate tectonic configurations, constraining the onset of the CNS and OAE1a, and correlations of terrestrial and marine carbon isotope responses to OAE1a during the Early Aptian.

In addition to Chron M0r, a brief reversed magnetic chron referred to as M”-1”r (*27*) or as ISEA [from the original site designation for level A at the Italian section ISE of Sentino (*28*)] occurred during the early Aptian (*29*), providing an independent tie-point for calibrating Chron M0r. However, the age of ISEA remains highly uncertain (*24*, *25*). Notably, the prevailing age estimates for both Chrons M0r and M”-1”r/ISEA are largely derived from magnetostratigraphic studies of Lower Cretaceous marine sequences or of single-site basaltic to intermediate lavas (*20*–*22*, *30*). Limited availability of reliable radiometric dates from marine records, combined with the discontinuous nature of magmatic rock units, likely exacerbates these chronological ambiguities. In this case, a direct drill hole from the terrestrial Lower Cretaceous with high-precision age determinations and nearly continuous sedimentary units could provide key constraints on the chronologies of Chron M0r, Chron M”-1”r/ISEA, and OAE1a.

In this study, we integrate high-resolution magnetostratigraphy and cyclostratigraphy to establish a high-precision temporal framework for the Yanshan Scientific Drilling Project (YSDP-4) drill core with a drill depth of 1497.5 m. This targeted the terrestrial Jiufotang Formation in the Kazuo Basin of the North China Craton (NCC), which is a key formation preserving fossils of the Jehol Biota, e.g., feathered dinosaurs and birds, and had previously yielded a ^40^Ar/^39^Ar date of 122.1 ± 0.3 Ma (*31*). We directly constrain the precise termination age of Chron M0r and determine a reliable age for the M”-1”r/ISEA magnetic chron and OAE1a. This recalibration offers a robust temporal framework to identify asynchronous carbon cycles between terrestrial and marine systems during OAE1a.

## RESULTS

### Geologic background and samples

**Fig. 1.**
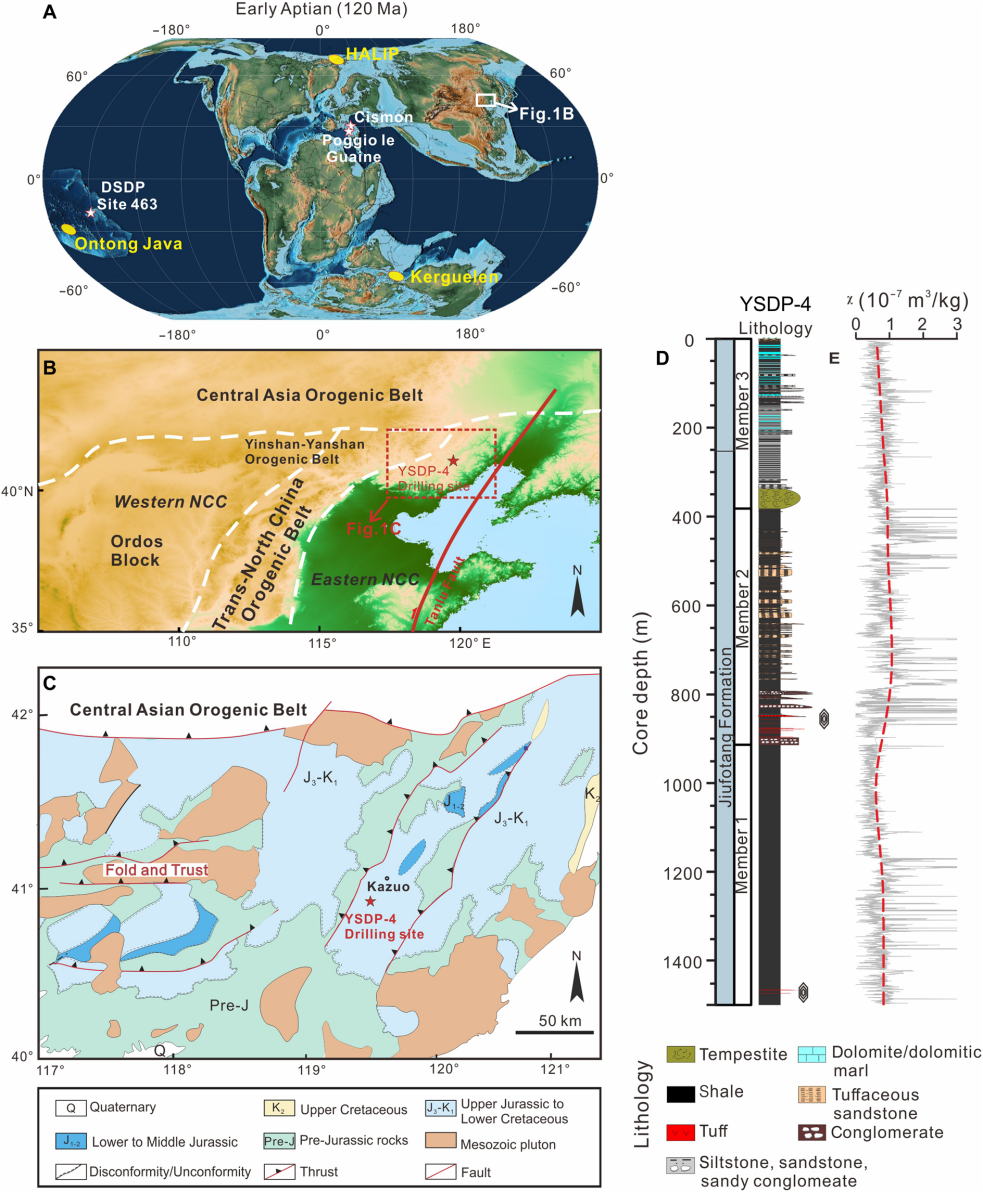
Geological map of the study region. (**A**) Paleogeographic map of the Early Aptian world (~120 Ma), Paleomap created using GPlates (*71*), with the plate rotation from Scotese and Wright (*72*). The stars filled with white color denote selected marine reference sites of the OAE1a, and the ellipses filled with yellow color represent the sites of Early Cretaceous LIPs. (**B**) Tectonic map of the NCC, showing the location of the YSDP-4 drill core. (**C**) Regional geological map of the northern margin of the NCC, modified from figure S2B in Hou *et al.* (*73*) (http://creativecommons.org/licenses/by/4.0/), showing the distributions of sedimentary units in the study region. (**D**) Stratigraphic column of the YSDP-4 drill core. Lithology follows Sun *et al.* (*35*). Dated zircon-bearing tuff layers are marked by diamonds at 1464.5 and 852 m in (D). (**E**) Peaks removed magnetic susceptibility (χ) data (gray line) alongside the LOESS detrending (red dashed line).

The NCC constitutes eastern and western blocks, which were welded together by the Trans-North China Orogenic belt during the early Paleoproterozoic (*32*) (Fig. 1, A and B). During the Mesozoic, the NCC underwent marked “destruction” by lithosphere thinning, which has been commonly attributed to paleo-Pacific plate subduction (*33*, *34*). This tectonic activity led to the development of numerous early Cretaceous rift basins within the NCC, including the Kazuo basin in western Liaoning Province (Fig. 1C). The YSDP-4 drilling hole (40.90255°N, 119.50293°E) in the Kazuo basin had targeted the entire Jiufotang Formation for drilling, but we did not penetrate its lower boundary, thus failing to reach the underlying Yixian Formation. Sedimentary lithology analyses indicate that this formation was deposited in a lacustrine environment (*35*). A continuous 1497.5-m-long core was retrieved with the predominant lithology of massive shales (Fig. 1D and fig. S1).

Stratigraphically, the borehole is subdivided into three members (Fig. 1D). Member 1 (1497.5 to 913 m) consists predominantly of massive and laminated black shales interbedded with thin tuff layers. Zircons from a tuff layer at the 1464.5 m depth were dated using chemical abrasion–isotope dilution–thermal ionization mass spectrometry (CA-ID-TIMS) U-Pb methods, yielding an age of 121.05 ± 0.32 Ma (*35*). Member 2 (913 to 382 m) comprises massive black shales interbedded with tuffaceous sandstones, including a 15-m-thick conglomerate layer and an 8-m-thick tuff layer in its base interval. Zircons from the tuff at 852 m yielded a CA-ID-TIMS U-Pb age of 117.359 ± 0.031 Ma (*35*). Member 3 (382 to 5 m) consists of tempestite, highly laminated siltstone, and massive gray shales interspersed with sandstone, siltstone, dolomitic marls, and dolomite.

A total of 2325 samples were collected for magnetostratigraphic analysis. Of these, 1524 samples were initially obtained from the entire depth interval of 1497.3 to 6 m at 1 m intervals above 1465 and 0.5 m intervals below. An additional 801 samples were subsequently collected at 1 cm resolution from the identified reversed-polarity horizons and their immediately adjacent upper and lower layers. Furthermore, 5889 samples for magnetic susceptibility (χ) analysis were retrieved at 0.25 m intervals across the 1497.3- to 5.5-m section (Fig. 1E), providing an average temporal resolution of approximately 1.5 kyr, based on the established chronology (121.05 to 117.36 Ma).

### Magnetostratigraphy and cyclostratigraphy

Detailed rock magnetic experiments document that magnetite is the main carrier of the characteristic remanent magnetization (ChRM) of the terrestrial Jiufotang Formation sediments from the YSDP-4 drill core (figs. S2 to S4). However, in some dark gray to black samples, the ChRMs are carried by magnetite and iron sulfides (most likely greigite) (figs. S2 to S4). According to the demagnetization behavior, one or two remanent components are identified (fig. S5). A total of 1779 of the 2325 paleomagnetic specimens exhibit reliable ChRM directions (data S1). The drilling core is unoriented; therefore, only the magnetic inclination data were used for magnetostratigraphy interpretations. Plate reconstructions place the NCC in the Northern Hemisphere during the Early Cretaceous (Fig. 1A). In this case, we interpret intervals with at least four consecutive samples exhibiting positive inclinations as normal polarity, and those with at least four consecutive samples displaying negative inclinations as reversed polarity. Isolated samples with negative inclinations that do not meet this criterion are therefore not considered in the magnetostratigraphic interpretation.

**Fig. 2.**
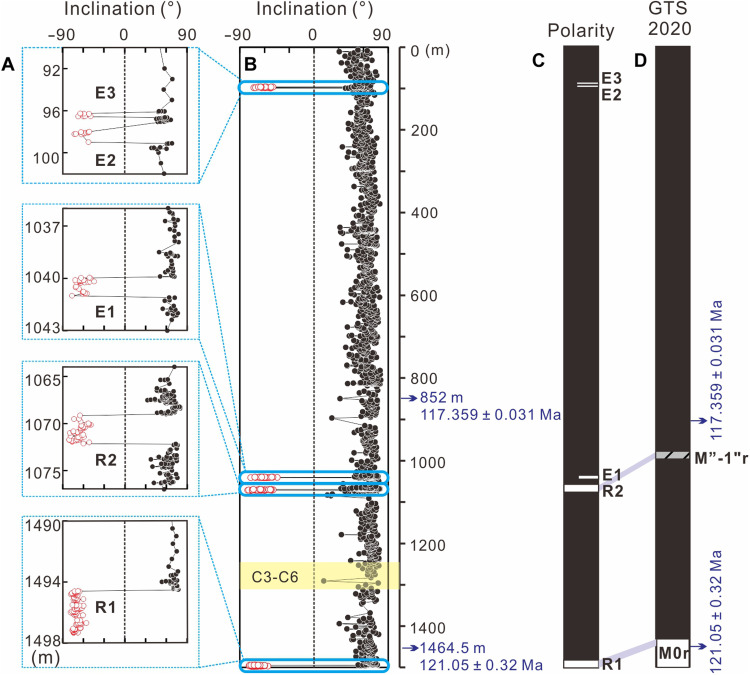
Magnetostratigraphy of the YSDP-4 drill core. (**A** and **B**) Magnetic inclination. The zoomed insets in (A) show details of the reversed-polarity intervals R1 and R2 and E1-E3. (**C**) Polarity zonation of the YSDP-4 core. Black indicates normal polarity, white bars represent reversed polarity, and half white bars denote geomagnetic excursions. (**D**) Reference geomagnetic polarity timescale (GTS 2020) (*25*). The C3-C6 interval of carbon isotope anomalies and radioisotopic dates from the core were also annotated in (B) and (D), respectively.

The results indicate only two narrow reversed-polarity zones (labeled R1 and R2) in the intervals of 1497.33 to 1494.60 m at the base of the core and of 1072.14 to 1069.16 m, respectively (Fig. 2, A and B). Considering that the zircons from the tuff layers at 1464.5 and 852 m yielded robust CA-ID-TIMS U-Pb ages of 121.05 ± 0.32 and 117.359 ± 0.031 Ma (*35*), respectively, we tentatively correlate the reversed polarity zones R1 and R2 with Chron M0r and Chron M”-1”r/ISEA, respectively, according to the geomagnetic polarity time scale in GTS 2020 [Fig. 2, C and D, and (*25*)]. An additional three, very narrow intervals with negative inclinations were observed at 1041 to 1039.97, 99 to 98, and 96.56 to 96.28 m (Fig. 2, A and B). Apart from these five intervals with negative inclinations, the remainder of the core is characterized by positive inclinations, indicative of normal polarity. A brief reversed-polarity zone, termed reversal “2,” has recently been proposed with an age of 116.17 ± 0.14 Ma based on orbital tuning of a short reversed geomagnetic polarity interval from DSDP Site 402 A (*36*). However, the 1041 to 1039.97 m interval corresponds to an astronomical tuning age of 118.38 Ma (data S2 and S3), ruling out any potential correlation with the reversal 2. Given that the synthesis in GTS 2020 does not include a second reversed-polarity zone after 117 Ma in the Aptian (*25*), the short intervals with negative inclinations at 99 to 98 and 96.56 to 96.28 m are not interpreted as reversed-polarity zones in this study, pending verification by future magnetostratigraphic investigations of contemporaneous marine or terrestrial records. Consequently, we provisionally interpret those three intervals with negative inclinations at 1041 to 1039.97 (E1), 99 to 98 (E2), and 96.56 to 96.28 m (E3) as records of potential geomagnetic excursions (Fig. 2, A to C).

**Fig. 3.**
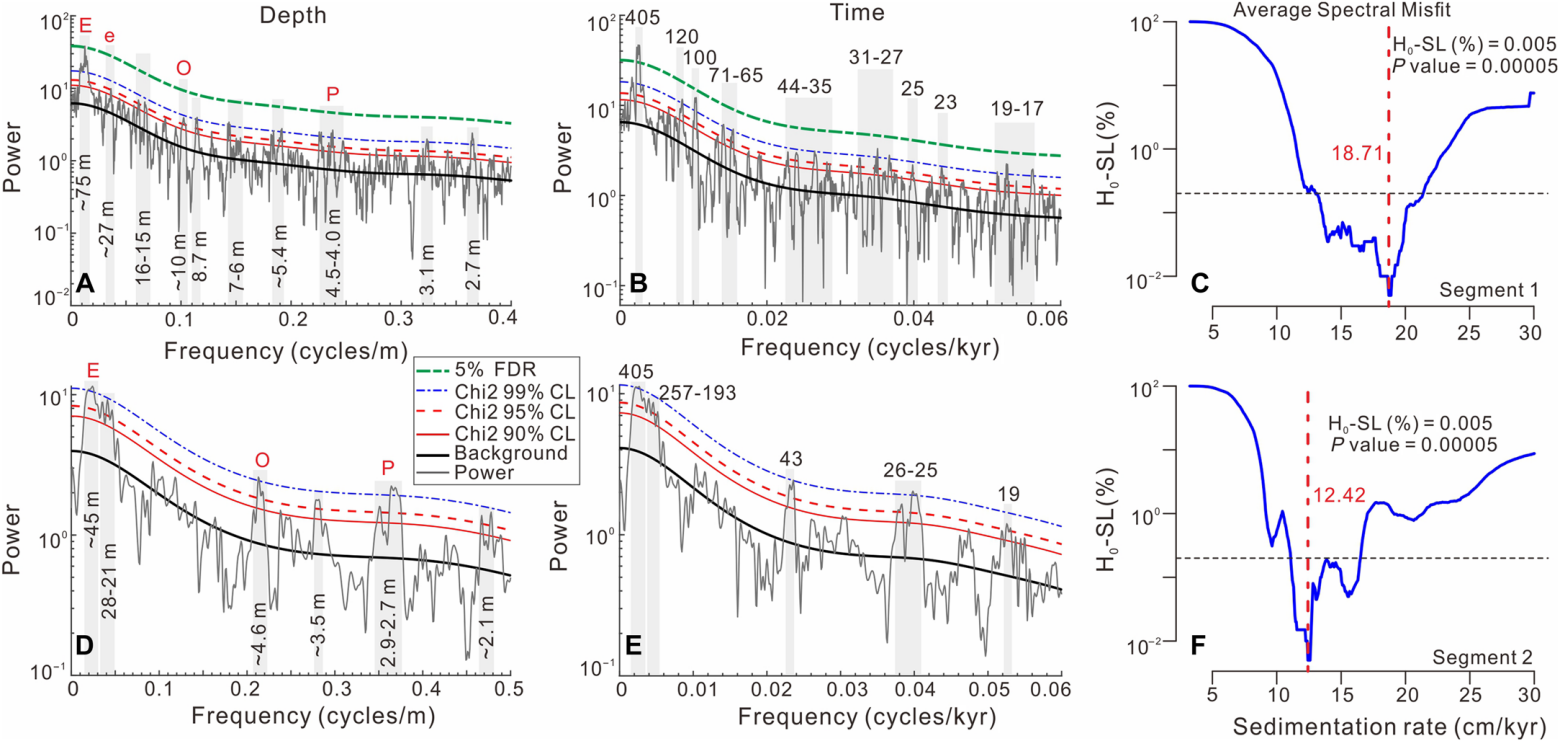
Spectral analyses and ASM results for the YSDP-4 Core. (**A** to **F**) 2π MTM power spectra of the depth-adjusted χ_fd_% dataset in depth (A and D) and time (B and E) domains, with corresponding ASM null hypothesis significance levels (C and F) for two segments. Notes: the spectral analyses were tested against a smoothed window average, and the false discovery rate (FDR) method procedure. Only peaks exceeding the χ^2^ 95% confidence level are labeled. CL, confidence level; E, long eccentricity; e, short eccentricity; O, obliquity; P, precession.

**Fig. 4.**
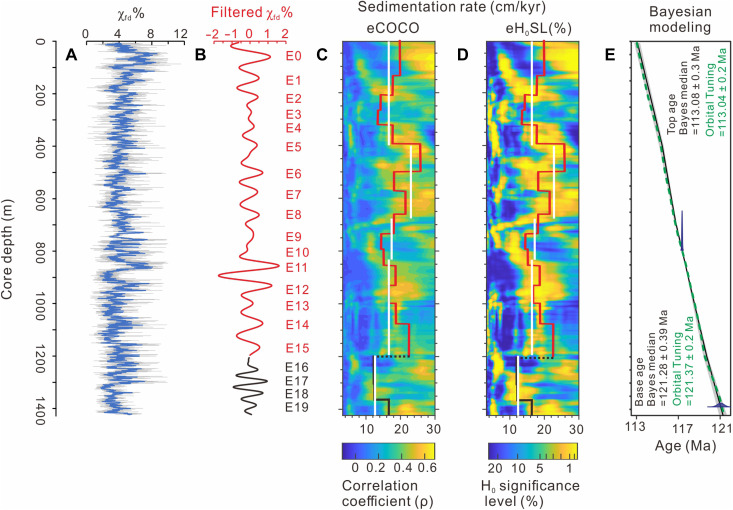
The reconstructed timeframe of the YSDP-4 drill core. (**A**) Depth-adjusted χ_fd_% series (gray curve), with a seven-point window moving mean (blue curve). (**B**) Filtered output of χ_fd_% series with approximately 75 (red line) and 45 m (black line) wavelengths (Gauss filter, passband: 0.0123 ± 0.005 and 0.0219 ± 0.006 cycles/m for 5.5 to 1200 and 1200 to 1423.25 m, respectively). (**C** and **D**) eCOCO sedimentation rate estimates using the χ_fd_% series. The sliding window is 120 m; the number of Monte Carlo simulations is 2000; sedimentation rate from 3.25 to 30 cm/kyr with a step of 0.05 cm/kyr. The red and black lines refer to sedimentation rates derived from the 405 kyr tuning, while the white line represents the median sedimentation rates derived from the astroBayes model. eH_0_SL denotes the evolutionary hypothesis significance level. (**E**) Age-depth model, constructed by Bayesian estimation integrated with astrochronology, radioisotopic geochronology, and astronomical tuning. The tuning age (green dashed line) is anchored to 117.359 ± 0.031 Ma (*35*). The black line, overlaid with light gray shading, represents the astroBayes model median with 95% credible interval. Dark blue colored probability distributions correspond to the radioisotopic dates reported in Sun *et al.* (*35*).

Before analysis, four thick intervals of ash (852 to 844 m), conglomerate (913 to 897 and 830 to 815 m), and tempestite (336 to 370 m) were excluded, yielding a remaining stratigraphic depth of 1423.25 m (data S3). The percentage of frequency-dependent magnetic susceptibility (χ_fd_%) was used as the primary cyclostratigraphic proxy for astronomical tuning. The rationale for this selection is provided in text S3. The depth-adjusted χ_fd_% series from the YSDP-4 core was divided into two segments based on evolutionary Fast Fourier transform (FFT) analysis (fig. S6): Segment 1 spans 5.5 to 1200 m (Fig. 3, A to C), and segment 2 spans 1200 to 1423.25 m (Fig. 3, D to F). Spectral analysis indicates that segment 1 displays prominent peaks at wavelengths of ~75, ~27, 16 to 15, ~10 to 5.4, and 4.5 to 2.7 m (Fig. 3A), whereas segment 2 is characterized by peaks at ~45, 28 to 21, ~4.6 to 3.5, and ~2.9 to 2.1 m (Fig. 3D). Sedimentation rates constrained by average spectral misfit (ASM), correlation coefficient (COCO), and TimeOpt analyses suggest that optimal values of 18.6 to 21.8 cm/kyr for segment 1 and 10.7 to 12.5 cm/kyr for segment 2 (Fig. 3, C and F, and figs. S7 to S9). On this basis, the ~75 and ~45 m wavelengths are interpreted as representing 405 kyr eccentricity cycles (Fig. 3, B and E); the ~27 and 16 to 15 m cycles in segment 1 correspond to 120 and 100 kyr eccentricity, while 28 to 21 m cycles in segment 2 do not align with known orbital cycles (Fig. 3, B and E); the ~10 to 5.4 m cycles in segment 1 and ~4.6 to 3.5 m cycles in segment 2 are attributed to obliquity, while 4.5 to 2.7 m cycles from segment 1 and 2.9 to 2.1 m cycles from segment 2 are interpreted as precession (Fig. 3, B and E). Accordingly, the depth-adjusted χ_fd_% series (Fig. 4A), bandpass filtered at ~75 and ~45 m wavelengths, were used to construct the age model, with each cycle was assigned a duration of 405 kyr (Fig. 4B). Sedimentation rates derived from 405-kyr filter vary from 12 to 26 cm/kyr throughout the core, with the trend consistently falling within the regions of high COCOs and statistical significance on the eCOCO maps (Fig. 4, C and D).

## DISCUSSION

### Chronology and duration of the Jiufotang Formation

The Jiufotang Formation, along with the underlying Yixian Formation, comprises lacustrine and terrestrial sedimentary strata preserving a crucial Jehol Biota record of diverse aquatic organisms and well-preserved early birds, feathered dinosaurs, pterosaurs, and primitive mammals (*37*). Extensive efforts have been made to date the Jiufotang Formation with the radiometric age constraints varying from 122.1 ± 0.3 to 118.9 ± 0.8 Ma (*31*, *38*). The uppermost Jiufotang Formation has been indirectly estimated to extend into the early Albian, based on a zircon U-Pb age of 112.6 ± 1.7 Ma from the overlying Shahai Formation (*39*). Astronomical tuning of the cyclostratigraphy by Sun *et al.* (*35*) constrains the upper boundary of the Jiufotang Formation to ~112 Ma, consistent within uncertainties in age estimates of the overlying Shahai Formation.

In this study, we construct a timescale for the YSDP-4 core using both classical astronomical tuning and the astroBayes method, the latter being a Bayesian inversion of astrochronology and radioisotope geochronology to derive an age-depth model (*40*). Orbital tuning constrains the base and top of the YSDP-4 core to 121.37 ± 0.2 and 113.04 ± 0.2 Ma (data S3), respectively, consistent within error with the astroBayes estimates of 121.28 ± 0.39 Ma (base) and 113.08 ± 0.3 Ma (top) (Fig. 4E and data S4). Associated uncertainty estimation for orbital tuning is detailed in text S4. Notably, astronomical tuning was anchored to a single high-precision radioisotopic date of 117.359 ± 0.031 Ma at 852 m (*35*). Within this framework, the stratigraphic position at original 1464.5 m (with adjusted depth of 1390.5 m) yields an astronomical tuning age of 121.17 ± 0.2 Ma, slightly older but identical to the radioisotopic date of 121.05 ± 0.32 Ma within error. In contrast, the astroBayes model directly incorporates two radioisotopic dates, yielding an estimate of 121.05 ± 0.35 Ma at the same stratigraphic level. In this case, the astroBayes age model is preferred in the following discussion sections.

On the basis of the astroBayes age model, the age of the lowermost recovered sediments of the Jiufotang Formation is 121.28 ± 0.39 Ma, and the age of the uppermost Jiufotang Formation is projected to be 113.08 ± 0.3 Ma, with a duration of 8.2 ± 0.49 Myr for the drilled interval. The ~0.8 Myr discrepancy between our top estimates and those from Sun *et al.* (*35*) may originate from differences in the filter bandpass selections during astronomical tuning using different datasets, but our ages for the lowermost drilled sediment coincide. Given that we did not drill through the Jiufotang Formation to reach the underlying Yixian Formation, a 122.1 ± 0.3 Ma age estimate is tentatively assigned as its base age, indicating a total duration of ~9 million years for this formation.

### Refining the ages of Chron M0r and the onset of the CNS

Given the age constraints defining the deposition of the Jiufotang Formation in the Early Cretaceous, it may serve as a key terrestrial candidate for recording the Barremian-Aptian boundary, one of the most poorly constrained stratigraphic boundaries after the breakup of Pangea (*41*). There is not yet a formal decision on the primary correlation markers or the location for the Global Boundary Stratotype Section and Point (GSSP) for this boundary. Unlike other Cretaceous boundaries defined by biostratigraphic correlations, the Barremian-Aptian boundary had been widely and informally placed at the base of magnetochron M0r (*42*). This placement was based on the global synchronicity of the geomagnetic field polarity reversals, thereby making magnetostratigraphic correlations among marine and terrestrial deposits throughout global settings feasible. However, revision of the ammonite biostratigraphy at potential GSSP sites has led the current Aptian working group of the International Subcommission on Cretaceous Stratigraphy of the IUGS’s ICS to now prefer a placement of the Barremian-Aptian boundary at the negative carbon-isotope spike at the base of OAE1a, which is about 0.8 Myr younger than the onset of Chron M0r (*18*).

**Fig. 5.**
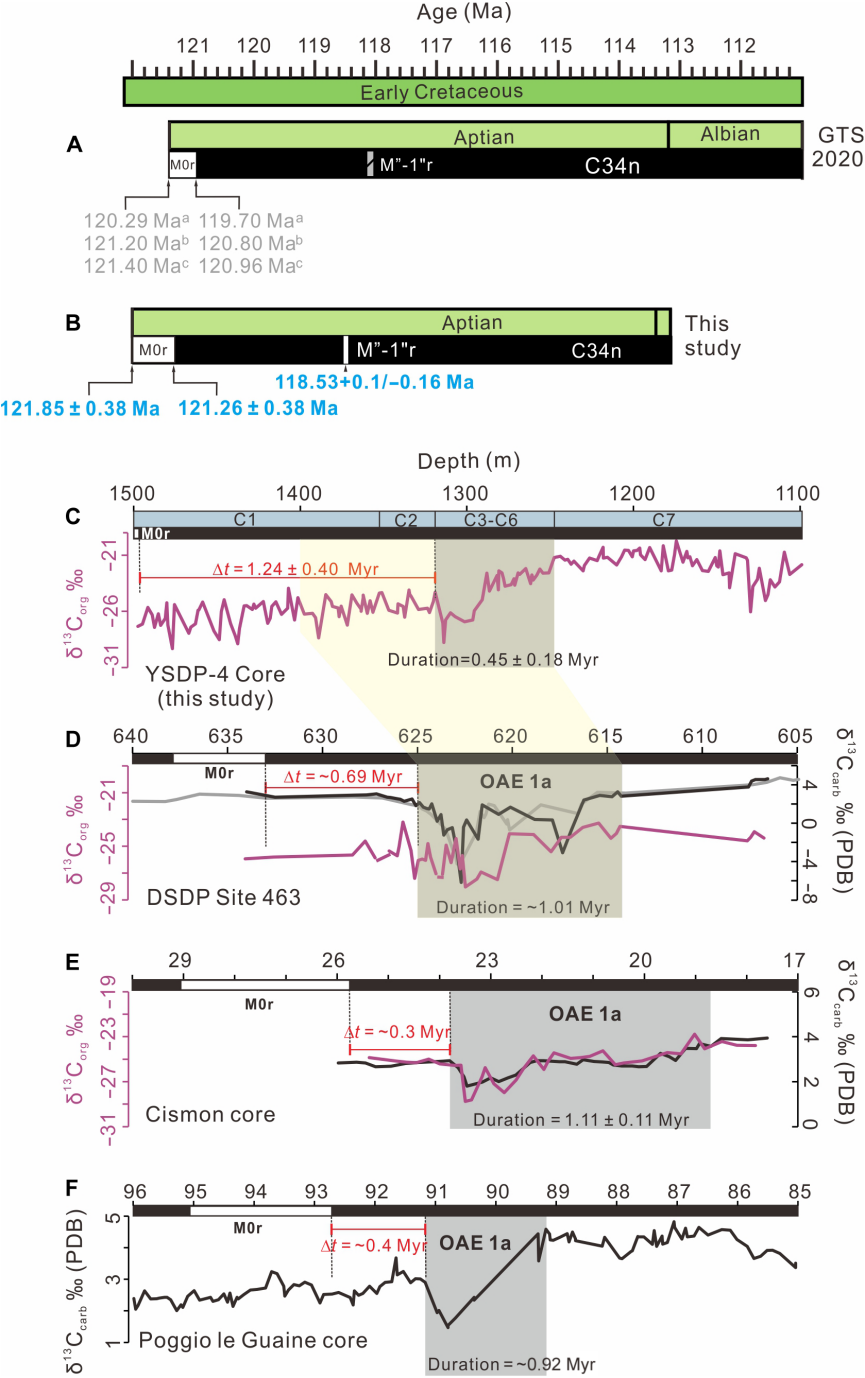
Comparisons of Aptian GTSs and carbon isotope profiles between marine and terrestrial sections. (**A**) GTS 2020 (*25*). “a, b, and c” indicate age constraints from Li *et al.* (*22*), Zhang *et al.* (*21*), and Ogg *et al.* (*25*), respectively. (**B**) Ages of Chron M0r and the brief reversed-polarity Chron M”-1”r/ISEA. (**C**) Organic carbon isotopic profile of YSDP-4 drill core (*35*), with subdivisions C1-C7 following Sun *et al.* (*35*). Δ*t* = 1.24 ± 0.40 Myr indicates that the onset of C3 interval in the YSDP-4 drill core lags behind the termination of Chron M0r by 1.24 ± 0.40 Myr. (**D** to **F**) Organic (brown line) and inorganic (gray and black lines) carbon isotopic profiles across the OAE1a interval from the DSDP Site 463, the Cismon core, and the Poggio le Gauine core. Data sources: black and brown lines in (D) and (E) from Bottini *et al.* (*8*); gray line in (D) from Ando *et al.* (*54*); black line in (F) from Leandro *et al.* (*16*). At DSDP Site 463 (D), the interval between the top (633.39 m) of Chron M0r and the OAE1a onset (625 m) in (D) is 8.39 m (*8*), corresponding to a Δ*t* = ~0.69 Myr, calculated using an average sedimentation rate of 12.1 m/Myr (*54*). In the Cismon core (E), the interval between the top (25.75 m) of Chron M0r and the OAE1a onset (23.67 m) is 2.08 m (*8*), corresponding to Δ*t* = ~0.3 Myr based on orbital tuning (*15*). In the Poggio le Guaine core (F), the interval between the top (92.7 m) of Chron M0r and the OAE1a onset (91.19 m) is 1.51 m (*16*), corresponding to Δ*t* = ~0.4 Myr from orbital tuning (*16*). The faint yellow bar across (C) and (D) marks the basal level of OAE1a.

The estimated age of Chron M0r has been considerably revised over time as new constraints emerged (Fig. 5A). Originally, Chron M0r and the past assignment of the Barremian-Aptian boundary was assigned an age of 121.0 ± 1.4 Ma, derived from consistency of whole-rock ^40^Ar/^39^Ar and zircon U-Pb dating of lavas from the MIT Guyot, OJP, and the Great Valley Group in California (*43*, *44*). This age is consistent with the M0r estimate of 121.2 ± 0.5 Ma, obtained in 2008 from reversely magnetized lavas in the Yixian Formation, Liaoning Province, northeast China (*20*). In contrast, GTS 2012 assigned it an age of 126.3 Ma (*24*) based on a cyclostratigraphic study of the Piobbico core from central Italy, which had suggested a duration of 13.42 Myr for the Aptian, and assuming a 99.6 Ma age for the Albian-Cenomanian boundary (*45*). It was later realized that there can be noticeable errors of over- or underidentification of eccentricity cycles in such a broad span without any precise radiometric ages to constrain the cyclostratigraphic interpretations (*19*).

In 2019, Olierook *et al.* (*19*) reexamined the available robust geochronological data from the Pacific Ocean, China, and California, as well as data from the Ontong Java Nui and High Arctic LIPs, and suggested that the age of the base of Chron M0r and the then-associated Barremian-Aptian boundary was between 123.8 and 121.8 Ma (*19*). This proposal is further supported by an interpolated age of 121.2 ± 0.4 Ma for the beginning of Chron M0r, based on magnetostratigraphic analysis of Chron M1r relative to U-Pb dates in the core DH1 from Svalbard, Norway (*21*). In contrast, a cyclostratigraphic study of the Poggio le Guaine core from Italy projected the Barremian-Aptian boundary at ~120.2 Ma, although no magnetostratigraphic and radiometric age data were provided for this estimate (*16*). Radiometric ages for tuffs recording a reversed-polarity interval in the Qingshan Group of the Jiaolai Basin (eastern China) (*22*) constrain the onset of Chron M0r to 120.29 ± 0.09 Ma, further corroborating this younger age model (*16*). However, a later study using high-precision ^40^Ar/^39^Ar dating of fresh sanidine collected from the rhyolite exposed in the Qingshan Group yielded an age of 121.71 ± 0.29 Ma (*46*), which the authors correlated with the stratigraphic position of the reversed magnetic polarity M0r to mark its onset (*46*).

The variation in the age of Chron M0r is primarily attributed to the lack of direct drill hole ties to high-resolution magnetostratigraphic and high-precision radiometric age data [e.g., (*20*, *22*)]. In this context, the YSDP-4 drill core provides invaluable opportunities for refining the age of Chron M0r and the onset of the CNS. The age model for the drill core also constrains the age for the later onset of C3 segment in the lacustrine setting, resembling C3 segment of OAE1a in marine records. As previously described, the YSDP-4 drill core reveals a magnetic reversed-polarity interval (R1) spanning from 1494.60 to 1497.33 m (Fig. 2A). The astroBayes model estimates the termination of this reversed-polarity interval (R1) at 121.26 ± 0.38 Ma (Fig. 5B and data S4). Notably, R1 occurs just below the negative δ^13^C_org_ excursion documented by Sun *et al.* (*35*) (Fig. 5C), which has been correlated with the δ^13^C_org_ excursion segments “C3-C6” associated with the OAE1a in marine archives (*2*). This correlation aligns with the stratigraphic position of magnetochron M0r below the OAE1a δ^13^C_carb_ excursion in global Aptian reference sections (*15*, *47*). Consequently, it is reasonable to correlate R1 with Chron M0r in the YSDP-4 drill core.

In the present study, the end of Chron M0r and the onset of CNS are estimated at 121.26 ± 0.38 Ma, which is ~0.3 Myr older than the estimate in the GTS 2020 (*25*), but within the uncertainty of both estimates. Considering that the duration of Chron M0r is estimated to be 0.59 ± 0.041 Myr (*48*), the onset of Chron M0r is thus projected at 121.85 ± 0.38 Ma (Fig. 5B), which is consistent within uncertainties with the estimates from He *et al.* (*20*) and Zhang *et al.* (*21*). However, it is important to note that Zhang *et al.* (*21*) did not provide magnetostratigraphic and radiometric age data to directly constrain the base of Chron M0r and the results from He *et al.* (*20*) were based on single-site igneous lavas, lacking direct constraints on either the base or the termination of Chron M0r. In summary, we place the base of Chron M0r at 121.85 ± 0.38 Ma and the onset of CNS at 121.26 ± 0.38 Ma (Fig. 5B), as inferred from integrated magnetostratigraphic and cyclostratigraphic results of YSDP-4 drill core from the terrestrial Jiufotang Formation in northeast China.

### Chronology of the brief reversed magnetic Chron M”-1”r/ISEA

A brief magnetic reversed-polarity interval known as M”-1”r/ISEA within the early part of the CNS has been identified since the 1970s in Deep Sea Drilling Project cores (e.g., DSDP Sites 317 A, 402 A, and 463) and in northern Italy (*29*, *49*). It occurs within the *Globigerinelloides algerianus* foraminiferal zone, but the precise chronologic age of Chron M”-1”r/ISEA is still unresolved, thus impeding its use as a reliable tie point for calibrating Chron M0r. Originally, M”-1”r/ISEA was given a vague estimated age of ~115 Ma (*29*). Gvirtzman *et al.* (*50*) reported an ^40^Ar/^39^Ar age of 118.0 ± 1.5 Ma for a basaltic unit with a geomagnetic polarity reversal from reversed to normal-polarity in Israel, interpreting this age as that of M”-1”r/ISEA. Zhu *et al.* (*30*) reported a ^40^Ar/^39^Ar age of 116.8 ± 3.0 Ma for lavas with reversed-polarity remanent magnetization from Liaoning Province, northeastern China, that was interpreted as Chron M”-1”r/ISEA. GTS 2012 had assigned a markedly older age of ~122 Ma for Chron M”-1”r/ISEA based on an Aptian cyclostratigraphy study (*24*), which was revised to a ~118 Ma estimate in GTS 2020 (*25*) based on reevaluation of that cyclostratigraphy. A recent study assigned the onset of Chron M”-1”r/ISEA to 117.03 ± 0.14 Ma using the orbital tuning method (*36*), despite the absence of robust radiometric age ties.

The age discrepancies in defining Chron M”-1”r/ISEA highlight the need for additional constraints to accurately calibrate the age for this brief reversed-polarity magnetochron. Our studied YSDP-4 drill core records a magnetic reversed-polarity interval (R2) spanning from 1072.14 to 1069.16 m (Fig. 2A), with an estimated astroBayes age of 118.53 +0.1/−0.16 Ma (Fig. 5B and data S4). In this context, we interpret R2 to correlate to Chron M”-1”r/ISEA of the GTS 2020. It is noteworthy that the estimated age of Chron M”-1”r/ISEA postdates the termination of Chron M0r defined in this study by 2.73 ± 0.41 Myr. This is within error of the estimated offset (~2.9 Myr) between Chrons M0r and M”-1”r/ISEA in GTS 2020 (*25*), further supporting our assignments of the recognized reversed-polarity intervals R1 and R2 in the YSDP-4 drill core to Chrons M0r and M”-1”r/ISEA, respectively.

### Asynchronous terrestrial and marine carbon isotope excursions relative to OAE1a

After Chron M0r, the most notable global event of the early Aptian was the OAE1a (*51*), which has often been associated with the emplacement of the OJP LIP (*10*, *52*, *53*). It is hypothesized that such a LIP event would rapidly release vast amounts of greenhouse gases (e.g., CO_2_) into the atmosphere and ocean systems, thereby triggering global warming, ocean acidification, and widespread marine biotic crises (*3*, *11*). A defining isotopic signal marking the onset of OAE1a is a pronounced NCIE during the C3 interval, reflecting a substantial injection of isotopically light carbon into marine carbon reservoirs (*8*, *10*, *12*). However, it remains uncertain whether terrestrial systems responded synchronously to this carbon cycle perturbation at the onset of OAE1a. Addressing this question requires not only precise chronological constraints on the initiation of OAE1a but also identification of comparable NCIEs in contemporaneous terrestrial archives. While marine records of OAE1a have been extensively studied, terrestrial evidence is sparse and less temporally constrained. In this context, the YSDP-4 drill core, which samples lacustrine deposits from the Jiufotang Formation in northeastern China, provides a valuable archive for assessing potential terrestrial responses to OAE1a.

Sun *et al.* (*35*) presented a carbon isotope stratigraphy for the YSDP-4 drill core, documenting a δ^13^C_org_ excursion from negative to positive at the depth interval of 1314.50 to 1247.60 m, resembling the δ^13^C_org_ variation trends of C3-C6 intervals in the marine sections (Fig. 5C) (*15*). The astroBayes age model developed in this study constrains the onset of C3 and the termination of C6 in the YSDP-4 drill core to 120.02 ± 0.13 and 119.57 ± 0.12 Ma, respectively, with a duration of 0.45 ± 0.18 Myr for the C3-C6 interval (Fig. 5C and data S4). This calibration places the time lag between the top of Chron M0r (with adjusted depth of 1420.6 m) and the onset of C3 interval (with adjusted depth of 1240.5 m) to 1.24 ± 0.4 Myr. However, the M0r-NCIE time interval in marine records is substantially shorter. At DSDP Site 463 in the central Pacific (*8*), the top of Chron M0r at 633.39 m below seafloor (mbsf) and the onset of NCIE at 625 mbsf give an M0r-NCIE time interval of ~0.69 Myr (Fig. 5D), derived from a local sedimentation rate of 12.1 m/Myr (*54*). In addition, astronomical tuning coupled with Bayesian modelling for the Cismon APTICORE in the southern Tethyan ocean (anchored to a 121 Ma base age of Chron M0r) constrains the onset age of OAE1a at 120.21 ± 0.04 Ma, with an estimated duration of 1.1 ± 0.11 Myr (*15*), yielding a M0r-NCIE time interval of ~0.3 Myr (Fig. 5E). This estimate match well with the ~0.4 Myr M0r-NCIE time interval from Poggio le Guaine core, also in the southern Tethyan margin (*16*, *55*) (Fig. 5F).

Our refined chronology of OAE1a reveals that the onset of the negative δ^13^C_org_ excursion in the YSDP-4 drill core postdates that of the DSDP Site 463 by up to 0.55 ± 0.40 Myr and that at the Cismon and Poggio le Guaine cores by up to 0.94 ± 0.40 and 0.84 ± 0.40 Myr, respectively. This temporal offset provides compelling evidence for asynchronous terrestrial and marine carbon isotope excursions relative to OAE1a. Notably, palynological data from the YSDP-4 drill core reveal a shift in terrestrial climate from cool and semiarid/semihumid to hot and dry conditions around 120 Ma (*56*), indicating that the terrestrial climatic warming occurred substantially later than the initial carbon cycle perturbation in the marine realm. This interpretation is further supported by reconstructed *p*CO_2_ trends across OAE1a from the Cau section, showing that *p*CO_2_ levels only rose gradually through the first part of OAE1a and by <1000 parts per million during the C3 segment (*7*). Together, these palynological and geochemical evidence suggest that atmospheric *p*CO_2_ remained relatively low at the onset of OAE1a, thereby challenging the hypothesis of rapid and large-scale emissions of greenhouse gases (e.g., CO_2_) into the atmosphere at the beginning of the OAE1a.

The observed different M0r-NCIE time intervals in terrestrial and marine records suggest that the OAE1a CIE did not occur synchronously across environments and locations, thereby calling into question the proposal of placing the Barremian-Aptian boundary at the base of OAE1a. Furthermore, this asynchrony highlights the spatial complexity of Earth’s carbon cycle and points to differing mechanisms and response times for carbon release, transport, and sequestration across environmental domains. These findings are critical for improving stratigraphic correlations between marine and terrestrial realms, advancing our understanding of global environmental change during the Early Aptian, and providing more boundary conditions for Earth system models used to predict carbon-climate feedbacks under future warming scenarios.

## MATERIALS AND METHODS

All the samples analyzed in this study were obtained from the YSDP-4 drill core, which is curated in a specialized repository at the Guangzhou Institute of Geochemistry, Chinese Academy of Sciences. The core is open to international scholars for visits and collaborative research.

### Paleomagnetism

To establish the magnetic polarity stratigraphy, paleomagnetic measurements were performed on 2325 specimens. The first set of 393 specimens underwent progressive thermal demagnetization in 24 steps, up to a maximum temperature of 680°C, with intervals of 30° to 50°C below 300°C, 25°C between 300° and 575°C, and 10°C above 575°C. Thermal demagnetization was conducted using the PGL-100 thermal demagnetizer with a residual field of <10 nT (*57*). The remanences were measured using a 2-G Enterprises Model 755 cryogenic magnetometer, installed in a magnetically shielded room (<300 nT). Data processing showed that 250 specimens yield reliable ChRM directions. In contrast, the second set of 1932 specimens was subjected to a composite scheme of alternating field demagnetization (up to 48 to 60 mT at intervals of 2 to 5 mT) followed by progressive thermal demagnetization (up to 420° to 500°C at intervals of 20° to 50°C). The remanences were measured using a 2-G Enterprises model 760 cryogenic magnetometer, housed in the same magnetically shielded room. This analysis obtained reliable ChRM directions from 1529 specimens. All magnetic measurements were conducted at the Paleomagnetism and Geochronology Laboratory, Institute of Geology and Geophysics, Chinese Academy of Sciences.

The ChRM of each specimen was effectively isolated using these two methods after removing the soft secondary component of magnetization through alternating field demagnetization above 15 mT or thermal demagnetization beyond 300°C. Demagnetization results were assessed by orthogonal diagrams (*58*), and the principal component directions were computed by PaleoMag software, applying the least-squares fitting technique (*59*, *60*). The criteria for selecting the demagnetization data for ChRM calculation were as follows: (i) at least four consecutive demagnetization points and (ii) the maximum angular deviation is less than 15°.

### Magnetic susceptibilities

A total of 5889 samples were analyzed for magnetic susceptibility analysis using the MFK-FA Kappabridge Magnetic Susceptibility System. We measured the low-frequency (976 Hz) magnetic susceptibility (χ_lf_) and high-frequency (3904 Hz) magnetic susceptibility (χ_hf_) with a magnetic field amplitude of 200 A/m. Samples were measured at each frequency three times, and the final χ_lf_ and χ_hf_ values were calculated by averaging the three measurements. The percentage of frequency-dependent magnetic susceptibility (χ_fd_%) was then calculated using the following equation: χ_fd_% = 100% × (χ_lf_ − χ_hf_)/χ_lf_.

### Cyclostratigraphy

Time-series analysis of the χ_fd_% dataset was performed with Acycle version 2.8 software and the Astrochron version 1.4 R package (*61*, *62*). Astronomical timescale (ATS) construction followed a multistep procedure: 

1) Data preparation: The depth-adjusted χ_fd_% series was segmented into two sections (5.5 to 1200 and 1200 to 1423.25 m) based on evolutionary FFT analysis (fig. S6). The segmented χ_fd_% series was further interpolated with a median sampling rate, and long-term non-Milankovitch trends were removed using robust locally weighted scatterplot smoothing (rLowess) (*63*).

2) Spectral analysis: The 2π multitaper method (MTM) was used to identify cyclicities (*64*), with background noise assessed by the Smoothed Window Averaging method (*65*). Statistical significance of spectral peaks was evaluated through a false discovery rate (*65*, *66*).

3) Sedimentation rate estimation: Sedimentation rates were constrained using three independent statistical tuning methods: ASM (*67*), COCO with its evolutionary extension (eCOCO) (*68*), and timescale optimization (TimeOpt) (*62*). ASM assesses misfit between observed spectral peaks and target orbital frequencies across a range of tested sedimentation rates, testing the null hypothesis of no orbital forcing (*67*). COCO quantifies COCOs between periodograms of an astronomical solution and the χ_fd_% series across trial rates (*68*); optimal rates maximize correlation, minimize H_0_-SL (no orbital forcing) significance (<1%), and incorporate the most orbital components (*68*). eCOCO extends COCO in sliding windows to track sedimentation-rate variations with depth (*68*). TimeOpt is an astronomical testing approach through an evaluation of eccentricity-related amplitude modulation and bundling (*62*).

4) Cycle identification: On the basis of detected χ_fd_% cycle ratios and the estimated optimal sedimentation rates, different periods were identified to represent long or short astronomical cycles. Long eccentricity cycles were segregated via Gauss bandpass filtering (passband: 0.0123 ± 0.005 and 0.0219 ± 0.006 cycles/m for 5.5 to 1200 m and 1200 to 1423.25 m, respectively) and are ultimately used for age model construction (*69*).

5) Tuning: The depth-adjusted χ_fd_% series was tuned to the time domain using the 405 kyr cycle tuning strategy, as it is the most stable orbital cycle over the past 250 Myr (*70*). The resulting floating ATS was subsequently anchored to the high-precision CA-ID-TIMS date of 117.359 ± 0.031 Ma at 852 m to establish an absolute chronological framework.

### Bayesian age estimates

Bayesian age-depth modeling was conducted using the astroBayes version 1.0 R package, which integrates stratigraphic position with numerical time via Bayesian inference (*40*). The astroBayes analysis incorporated the following inputs: (i) the depth-adjusted χ_fd_% series as the cyclostratigraphic record; (ii) target orbital frequencies (eccentricity: 0.4057, 0.1307, 0.1238, and 0.0989; obliquity: 0.0386; precession: 0.02288, 0.02166, 0.01858, and 0.01844) calculated with the Milankovitch Calculator in Acycle version 2.8 at a reference age of 117 Ma; (iii) five stratigraphic boundary positions identified from evolutionary FFT analyses where frequency patterns remain stable (table S1); and (iv) two independent radioisotopic dates of 121.05 Ma (with adjusted depth of 1390.5 m) and 117.359 Ma (with adjusted depth of 794.5 m) in the YSDP-4 core. For each stratigraphic layer, a vague uniform prior distribution was specified, allowing sedimentation rate to take any value between specified minimum and maximum values (*40*) (table S1). This specification explicitly accounts for variability in sedimentation within the Bayesian framework (fig. S10). Posterior probability distributions were then used to estimate ages and associated uncertainties, yielding a median age model with 95% confidence intervals, thus capturing uncertainties from sedimentation rates, astronomical solutions, and radioisotopic constraints (*40*). Annotated R scripts used in these analyses are provided in text S5.

## Supplementary Materials

### The PDF file includes:

Supplementary Text Figs. S1 to S10 Legends for data S1 to S4 Table S1 References
