# Supplementary material for: Refined chronologies of magnetochron M0r reveal asynchronous terrestrial and marine carbon isotope responses to Oceanic Anoxic Event 1a
Source: Sci Adv. 2026 Mar 4;12(10):eaea8374. doi: 10.1126/sciadv.aea8374 (PMC12959394; doi:10.1126/sciadv.aea8374)
Supplement: Supplementary file 1 — Supplementary Text Figs. S1 to S10 Legends for data S1 to S4 Table S1 References [file sciadv.aea8374_sm.pdf]

Science Advances
AAAS

Supplementary Materials for

Refined chronologies of magnetochron M0r reveal asynchronous terrestrial and marine carbon isotope responses to Oceanic Anoxic Event 1a

Gui-Mei Lu *et al.*

Corresponding author: Cheng-Long Deng, cldeng@mail.iggcas.ac.cn; Yi-Gang Xu, yigangxu@gig.ac.cn

Sci. Adv. **12**, eaea8374 (2026)
DOI: 10.1126/sciadv.aea8374

The PDF file includes:

Supplementary Text
Figs. S1 to S10
Legends for data S1 to S4
Table S1
References

Other Supplementary Material for this manuscript includes the following:

Data S1 to S4

Supplementary Text

1. Rock magnetic methods

To identify the magnetic mineralogy and remanence carriers of the terrestrial deposits of the Jiufotang Formation from the YSDP-4 drill core, we conducted detailed rock magnetic analyses on representative samples of three types of sediments: Type I samples consist of grayish-white to gray siltstone; Type II samples, grayish-black argillaceous siltstone to mudstone; and Type III samples, dark gray to black mudstone. Figures S2 to S4 show the rock magnetic results for each type of representative samples.

Rock magnetic measurements in this study include temperature-dependence of magnetic susceptibilities (χ–T curves), hysteresis loops, isothermal remanent magnetization (IRM) acquisition and its back-field demagnetization of the saturation IRM (SIRM), first-order reversal curve (FORC) diagrams (*74, 75*), and progressive thermal demagnetization of three-axis IRM (*76*).

The χ–T curves were measured from room temperature to 700°C and back to room temperature using the Kappabridge MFK1-FA system with a CS-3 high-temperature furnace (AGICO Ltd., Czech Republic, sensitivity: 1×10^{-8} SI) in an argon environment.

Hysteresis loops, IRMs, and FORCs were measured at room temperature using a Princeton Measurements Corporation vibrating sample magnetometer (MicroMag VSM 3900). Each sample was measured up to a maximum field of 1.5 T; however, some were cut off at 0.5 T for clarity when plotting the loops. FORC analyses were performed using FORCinel version 1.18 software (*77*). The IRM component analysis was based on cumulative log-Gaussian analysis (*78–81*).

The S-ratio is defined as the ratio of IRM acquired at -0.3 T ($IRM_{-0.3T}$) to the IRM acquired at 1.5 T ($IRM_{1.5T}$, hereafter termed SIRM), i.e., S-ratio = - ($IRM_{-0.3T}$/SIRM). The $IRM_{0.1T}$/SIRM ratio was calculated as the ratio of the IRM obtained at 0.1 T ($IRM_{0.1T}$) to the SIRM, reflecting the relative contributions of low-coercivity components (*82*).

To characterize the remanence carriers and their blocking temperatures, we employed stepwise thermal demagnetization of three orthogonal component IRM (*76*). Specimens were sequentially magnetized in direct current fields of 2.50, 0.50, and 0.05 T along three

mutually orthogonal Z, Y, and X axes using a pulse magnetizer. Subsequently, these specimens were thermally demagnetized from room temperature to 680°C with 10–50°C intervals (22 steps).

2. Rock magnetic results

2.1 χ–*T* curves

The heating curves for three types of samples show a sharp decline in magnetic susceptibility around 550–585°C, with values approaching zero at approximately 585°C (fig. S2), indicating the presence of magnetite in all samples (*83*). Furthermore, the cooling curves are significantly higher than the heating curves, suggesting the neoformation of strongly magnetic minerals during the thermal treatment (*84*). Notably, the heating curves of Type III samples (figs. S2 G–I and G1–I1) exhibit a marked increase in magnetic susceptibility starting at 400°C, with a distinct peak around 500°C. The cooling curves for these samples are significantly higher than the heating curves, a phenomenon more pronounced than in Type I and Type II samples (figs. S2 A–F1). Although Type I samples also display a small peak in the heating curves around 500°C (figs. S2 A–B and A1–B1), it is not prominent within the overall range of magnetic susceptibility changes. This enhanced susceptibility in Type III samples is attributed to the extensive neoformation of magnetite grains from the transformation of iron-sulfide minerals such as greigite (*85, 86*), indicating that Type III samples contain not only magnetite but also iron-sulfide minerals such as greigite.

2.2 The three-axis IRM thermal demagnetization

Magnetic minerals with different coercivities can be distinguished based on their individual unblocking temperatures. Therefore, thermal demagnetization of the three-axis IRM (*77*) is a useful method for identifying the magnetic mineral assemblage (*83, 87*).

The three-axis IRM thermal demagnetization results show that low- and medium-coercivity IRM fractions are dominant in all samples (fig. S3). All components of different coercivities approach zero at approximately 580°C, with a maximum unblocking temperature of ~580°C. The behaviors indicate the dominance of magnetite as the remanence carriers and suggest negligible high-coercivity components.

For Type III samples, all three components exhibit a distinct two-stage decrease. The first stage is from ~240°C to ~350°C, where greigite begins to undergo chemical decomposition and a possible blocking temperature is observed (*88, 89*). The second stage continues from ~350°C to ~580°C, after which the remanence values drop to nearly zero (figs. S3 I−L). These behaviors indicate the coexistence of magnetite and greigite in the studied samples.

2.3 IRM

IRM acquisition curves and coercivity spectra analysis effectively capture the coercivity distribution of magnetic minerals in the samples (e.g., *90*).

As shown in fig. S4, over 80% of the SIRM is acquired by approximately 100 mT, with $IRM_{0.1T}$/SIRM > 0.81 and S-ratio > 0.96, indicating the dominance of low-coercivity ferrimagnetic minerals. However, the Bcr values for Type III samples are clearly higher than those of the other two types. The IRM spectral analyses (figs. S4 D–F) show that two distinct components can be identified. The low-coercivity component (IRM1), with coercivities between 10 and 40 mT, is consistently present and aligns with the coercivity of magnetite. In contrast, the high-coercivity component (IRM2) in Type III samples exhibits a slightly higher coercivity, exceeding 60 mT (fig. S4 F), which is consistent with the coercivity of greigite. Thus, Type III samples contain both magnetite and greigite, whereas the other two types contain only magnetite.

2.4 Hysteresis loops and FORC diagrams

Hysteresis loops and FORC diagrams provide further insights into the grain size of magnetic minerals and the interactions between magnetic particles (*75, 82, 86, 91*).

The hysteresis loops of all samples (figs. S4 G–I) close above 0.25 T, confirming the dominance of low-coercivity ferrimagnetic minerals. These hysteresis loops gradually widen from Type I to Type III samples. For Type III, they exhibit a slightly rectangular shape similar to that of single-domain (SD) particles, indicating finer grain sizes (fig. S4 I). This trend is more clearly observed in the FORC diagrams (figs. S4 J–L). Type I samples display a typical multi-domain (MD) state (fig. S4 J). Type II samples show characteristics of pseudo-single-domain (PSD) state (fig. S4 K), featuring concentric closed-contour

patterns along the Hu = 0 horizontal axis, resembling SD grains, and truncated, vertically extended patterns along the Hc = 0 vertical axis, also typical of MD grain characteristics.

In contrast, the FORC diagrams for Type III samples (fig. S4 L) exhibit a distinct "bull's-eye" pattern, which is characteristic of greigite (*82*). Additionally, the diagrams show a central contour with a slight deviation toward negative Bu values and a negative region, further indicating the presence of SD greigite (*82*), possibly of biogenic origin (*75*). This is consistent with the fact that greigite typically forms in an SD state in natural environments (*85, 92*). These results suggest that the magnetic minerals in Type III samples consist of finer PSD-SD-like magnetite and greigite.

2.5 Summary of the rock magnetic results

The detailed rock magnetic results detailed above document that in the samples of Type I (grayish-white to gray siltstone) and Type II (grayish-black argillaceous siltstone to mudstone), the magnetic characteristics are defined by a maximum unblocking temperature of approximately 580°C and are dominated by a single component of medium-low coercivity. These features indicate that magnetite is the primary carrier of magnetic remanence. Further analysis using FORC diagrams, hysteresis loops, and Day plot confirms that the remanence carriers in Type I and Type II samples are relatively coarse, PSD to MD-like magnetite grains.

While Type III (dark gray to black mudstone) samples share the same maximum unblocking temperature of around 580°C with Type I and Type II samples. Notably, Type III samples also exhibit a significant increase in magnetic susceptibility after 400°C, reaching hundreds to thousands of times the room-temperature magnetic susceptibility. This, combined with the characteristic FORC patterns of iron sulfides and a two-component coercivity distribution, indicates that in Type III samples, the remanence carriers are relatively fine-grained, PSD to SD magnetite and greigite.

In summary, detailed rock magnetic experiments demonstrate that magnetite is the main carrier of ChRM in the terrestrial Jiufotang Formation from the YSDP-4 drill core. The remanence carriers in Type I and Type II samples are relatively coarse, PSD to MD-like magnetite grains; while in Type III samples, the ChRM is carried jointly by relatively fine-grained, PSD to SD magnetite and greigite.

3. The rationale for the cyclostratigraphic proxy chosen

Magnetic susceptibility (χ) quantifies how easily a material can be magnetized in an applied magnetic field. It can vary with the frequency of the applied magnetic field. The frequency-dependence of magnetic susceptibility is expressed as the difference in magnetic susceptibility measured at low and high frequencies (χ_{fd}) or the percentage of frequency-dependent magnetic susceptibility ($\chi_{fd}\%$):

$$\chi_{fd} = \chi_{lf} - \chi_{hf}\ [10^{-8}m^3kg^{-1}] \quad (1)$$

$$\chi_{fd}\% = 100 \times (\chi_{lf} - \chi_{hf})/\chi_{lf}\ [\%] \quad (2)$$

The two proxies, χ_{fd} and $\chi_{fd}\%$, are often used to quantify ultrafine (near the superparamagnetic/stable single-domain (SP/SSD) boundary) ferrimagnetic particles (*93, 94*), which are commonly thought to represent the so-called "pedogenic magnetic fraction" (*94, 95*). Detailed rock magnetic measurements reveal the presence of a mixture of magnetite and greigite in the samples of the studied YSDP-4 drill core. Detrital magnetite from the adjacent source areas makes dominant contributions to the magnetic susceptibility, with subordinate contributions from penecontemporaneous greigite. Enhanced pedogenesis or chemical weathering, which is linked to warm/humid periods, promotes the formation of SP magnetite grains and consequently increases $\chi_{fd}\%$ values (*93, 96, 97*); Conversely, weakened pedogenesis or chemical weathering, which is linked to cold/dry periods, is unfavorable for the formation of SP magnetite grains and consequently shows lower values of $\chi_{fd}\%$ (*93, 96, 97*). However, the amplitude of χ_{fd} fluctuation does not necessarily reflect the amplitude of climate due to the potential changes in magnetic grain input from source areas. In contrast, the χ_{fd} normalized by low-frequency magnetic susceptibility, that is $\chi_{fd}\%$, can more faithfully reflect the amplitude of climate. For these reasons, we select $\chi_{fd}\%$ as the primary proxy for spectral analysis in this study.

4. Uncertainty of the astronomical time scale

For the astronomical tuning (ATS), we consider two major sources of uncertainty: (i) an uncertainty of 0.031 Myr from the radiometric dating of the tuff layer at 852 m (*35*), and (ii) the phase uncertainty associated with the 405-kyr eccentricity cycle used for tuning. The latter has been estimated in previous studies at 0.10–0.20 Myr (e.g., refs. *98, 99*),

reflecting a conservative and widely accepted approximation of the phase lag inherent in deep-time astronomical tuning. This lag may arise from delayed climate responses, variations in proxy recording, or local sedimentation dynamics. To remain conservative, we adopt an empirical phase uncertainty of 0.20 Myr. When combined in quadrature with the radiometric uncertainty, the overall uncertainty of our ATS is ~0.20 Myr, calculated as $\sqrt{0.031^2 + 0.2^2}$.

5. R scripts used for Bayesian age estimates.

```
library(astroBayes)
library(readr)
library(tidyverse)
library(ggplot2)
d1 <- read.csv("C:/Users/17336/Desktop/data/radioisotopic_dates.csv")
c2 <-  read.csv("C:/Users/17336/Desktop/data/MS_new.csv")
f1 <- read.csv("C:/Users/17336/Desktop/data/target_frequencies.csv")
l1 <- read.csv("C:/Users/17336/Desktop/data/layer_boundaries.csv")
age_model <- astro_bayes_model(geochron_data = d1,cyclostrat_data = c2,
target_frequency = f1, layer_boundaries = l1, iterations = 10000, burn = 1000)
n=2000
ridges <- age_model$geochron_data %>% mutate(low = age - age_sd * 4, high = age +
age_sd * 4) %>%
uncount(n,.id = 'row') %>%
mutate(x = (1 - row/n) * low + row/n*high,density = dnorm(x, age, age_sd))
data=fortify(age_model$CI, melt=TRUE)
ridges %>%
ggplot(mapping = aes(x = x, y = position,height = density,group = id,fill = id)) +
geom_density_ridges(stat = 'identity',scale = 0.25,color = NA,alpha = 0.75,
show.legend=FALSE) +ylab('Depth (m)') +xlab('Age (Ma)') +
scale_y_reverse(expand=c(0,0),limits = c(1459,5.5),breaks = seq(0,1400,50))+
geom_ribbon(data=data,mapping = aes(xmin = CI_2.5,xmax = CI_97.5,y =
position),inherit.aes = FALSE,alpha =
0.25)+geom_line(data=data,mapping=aes(y=position,x=median),inherit.aes = FALSE,
color="black", linewidth=0.5) +
scale_fill_viridis(discrete = TRUE, option = 'plasma', end = 0.9)+
scale_x_continuous(expand=c(0,0),limits = c(112.5,122), breaks = seq(112,122,0.5))+
theme_bw()
ggsave("age_depth.pdf", height = 5, width =2.8)
```

Supplementary Figures

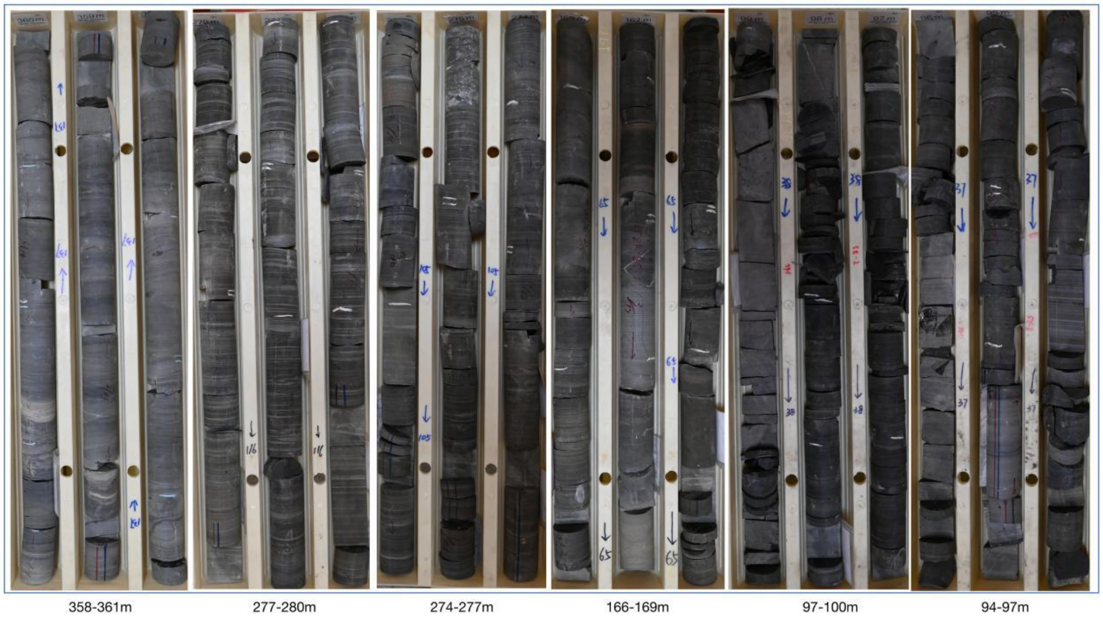

Fig. S1. Photographs of the YSDP-4 drill core from the terrestrial Jiufotang Formation in the Kazuo Basin, North China Craton.

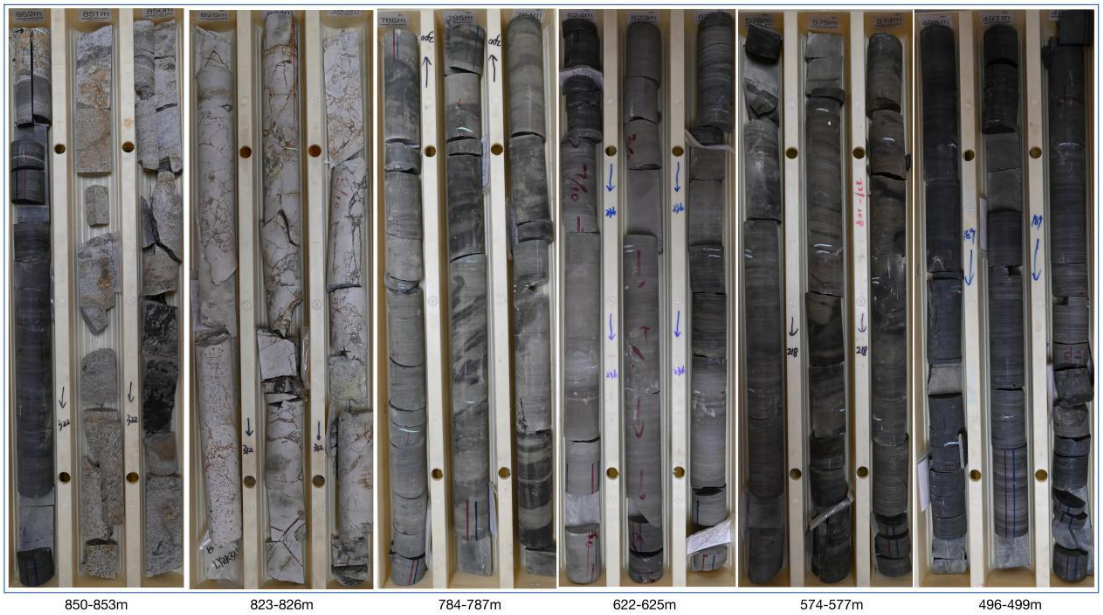

Fig. S1. Continued

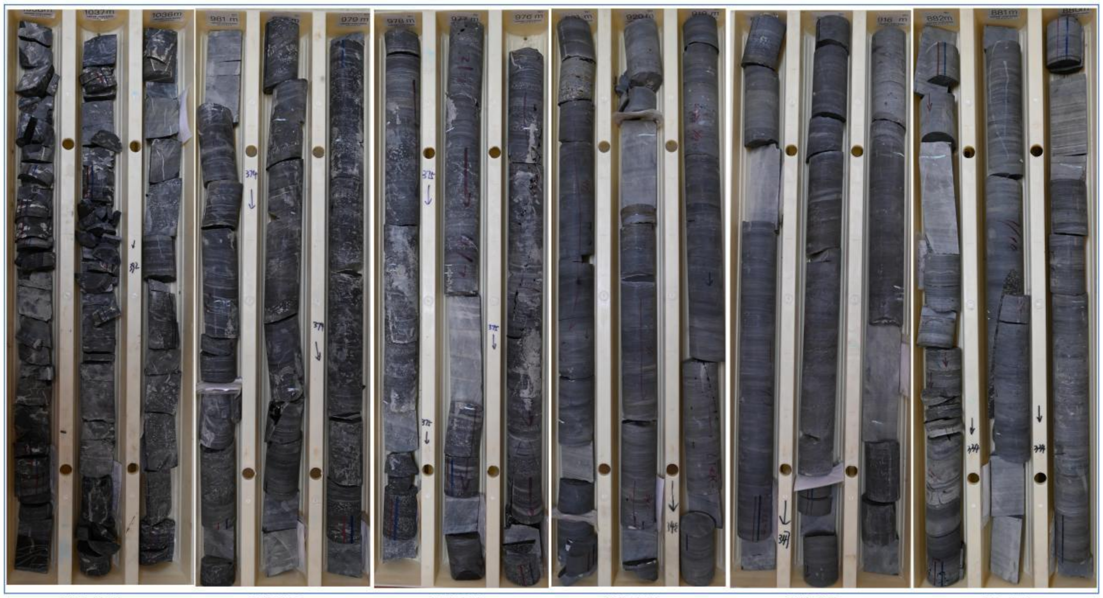

1036-1039m 979-982m 976-979m 919-922m 916-919m 880-883m

Fig. S1. Continued

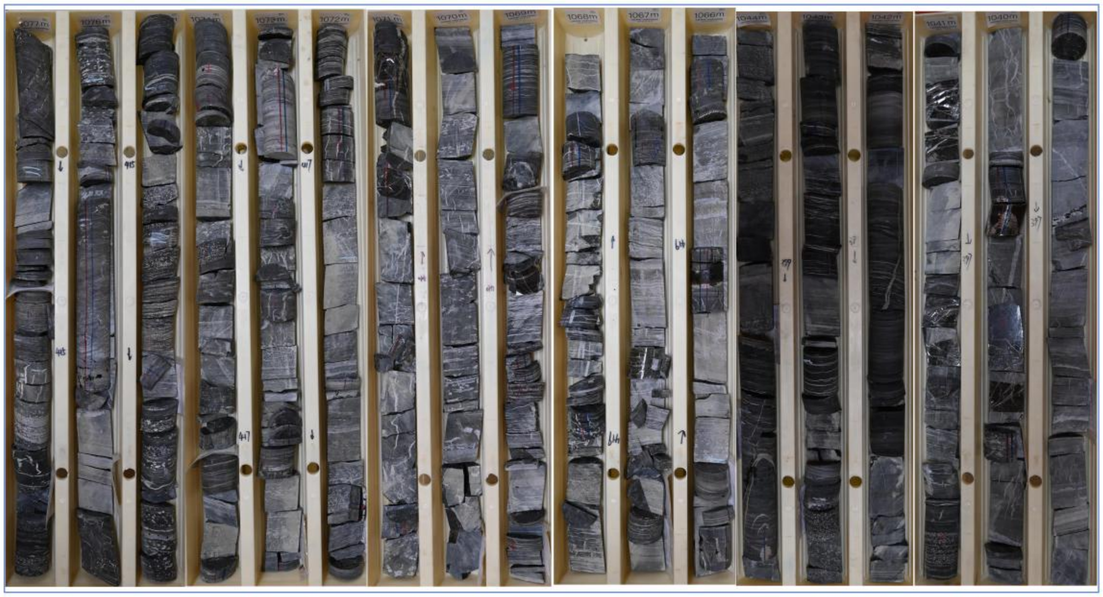

1075-1078m 1072-1075m 1069-1072m 1066-1069m 1042-1045m 1039-1042m

Fig. S1. Continued

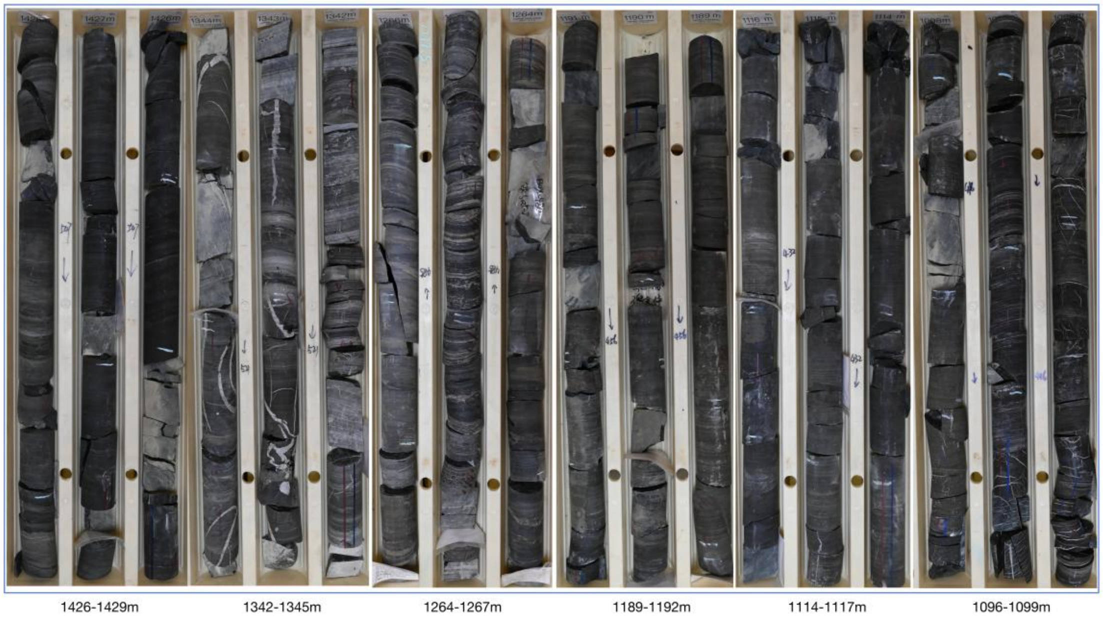

Fig. S1. Continued

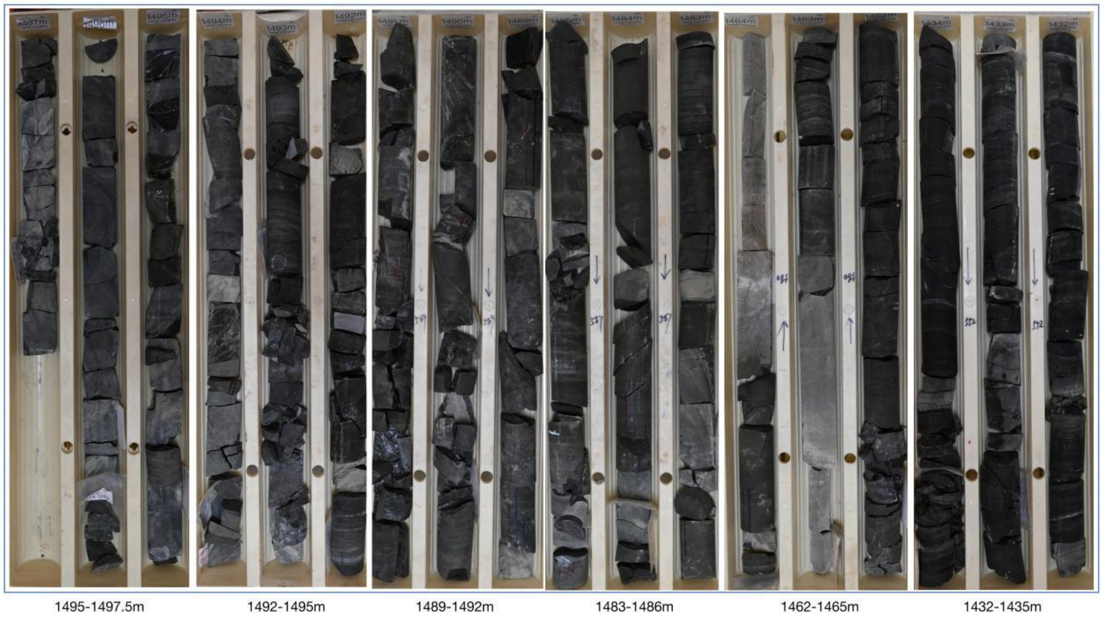

Fig. S1. Continued

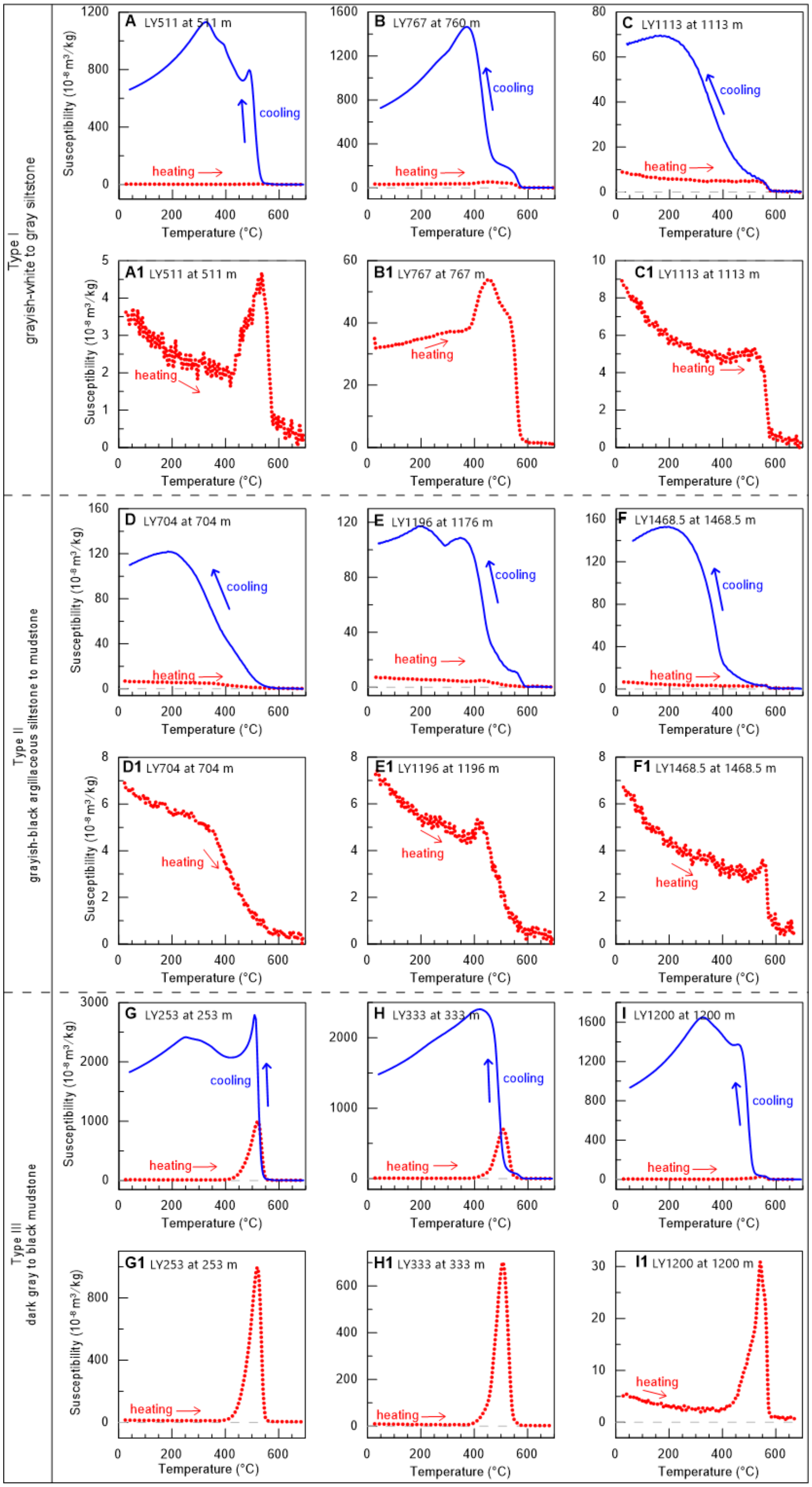

Fig. S2. χ**−*****T*** **curves of three types of representative samples from the terrestrial Jiufotang Formation of the YSDP-4 drill core**. Dotted and solid lines represent heating

and cooling curves, respectively. (**A–C**, **A1–C1**) Type I samples consisting of grayish-white to gray siltstone. (**D–F**, **D1–F1**) Type II samples consisting of grayish-black argillaceous siltstone to mudstone. (**G–I**, **G1–I1**) Type III samples consisting of dark gray to black mudstone.

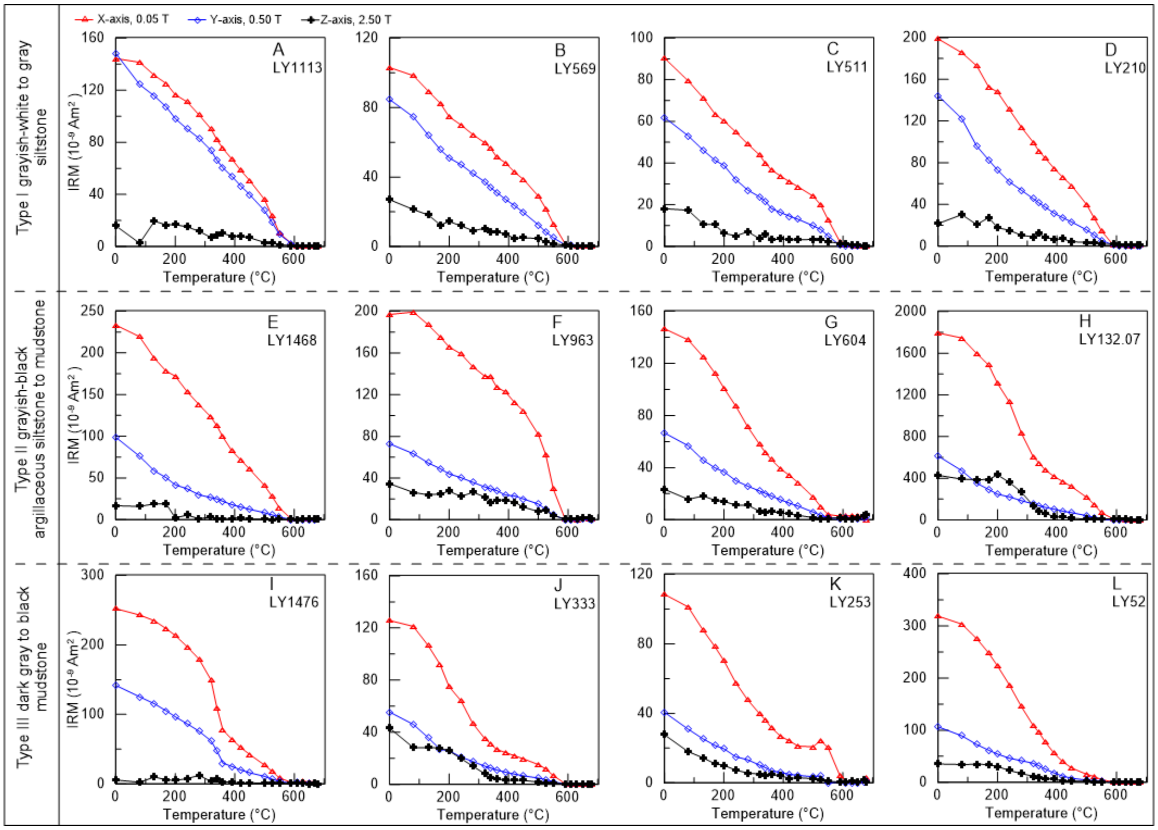

Fig. S3. Progressive thermal demagnetization of three-axis isothermal remanent magnetization (IRM) for three types of representative samples from the terrestrial Jiufotang Formation of the YSDP-4 drill core. (**A-D**) Type I samples consisting of grayish-white to gray siltstone; (**E-H**) Type II samples, grayish-black argillaceous siltstone to mudstone; and (**I-L**) Type III samples, dark gray to black mudstone.

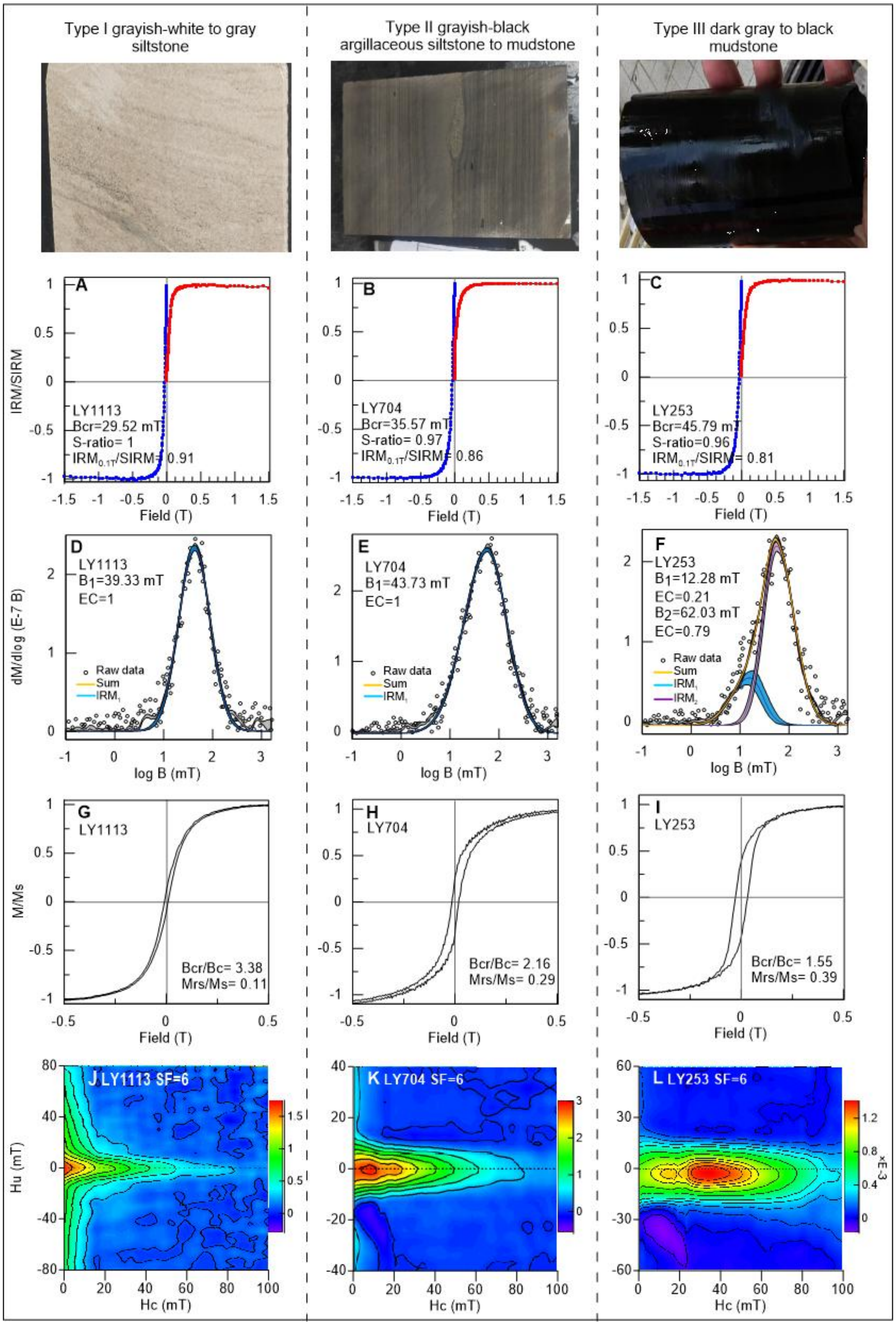

Fig. S4. Room-temperature rock magnetic results for three types of representative samples from the terrestrial Jiufotang Formation of the YSDP-4 drill core. Left panel,

type I sample of grayish-white to gray siltstone; central panel, type II sample of grayish-black argillaceous siltstone to mudstone; and right panel, Type III sample of dark gray to black mudstone. (**A–C**) IRM acquisition curves. (**D–F**) Component analyses of coercivity distributions with the shaded areas representing the 95% confidence intervals. Green and purple lines represent the fitted components 1 (IRM_1) and 2 (IRM_2), respectively. Yellow lines represent the sum of the model components. Grey represents the raw data. EC represents extrapolated contribution. (**G–I**) Hysteresis loops measured up to ±1.5 T and corrected for paramagnetic contributions. (**J–L**) FORC diagrams. SF, smoothing factor.

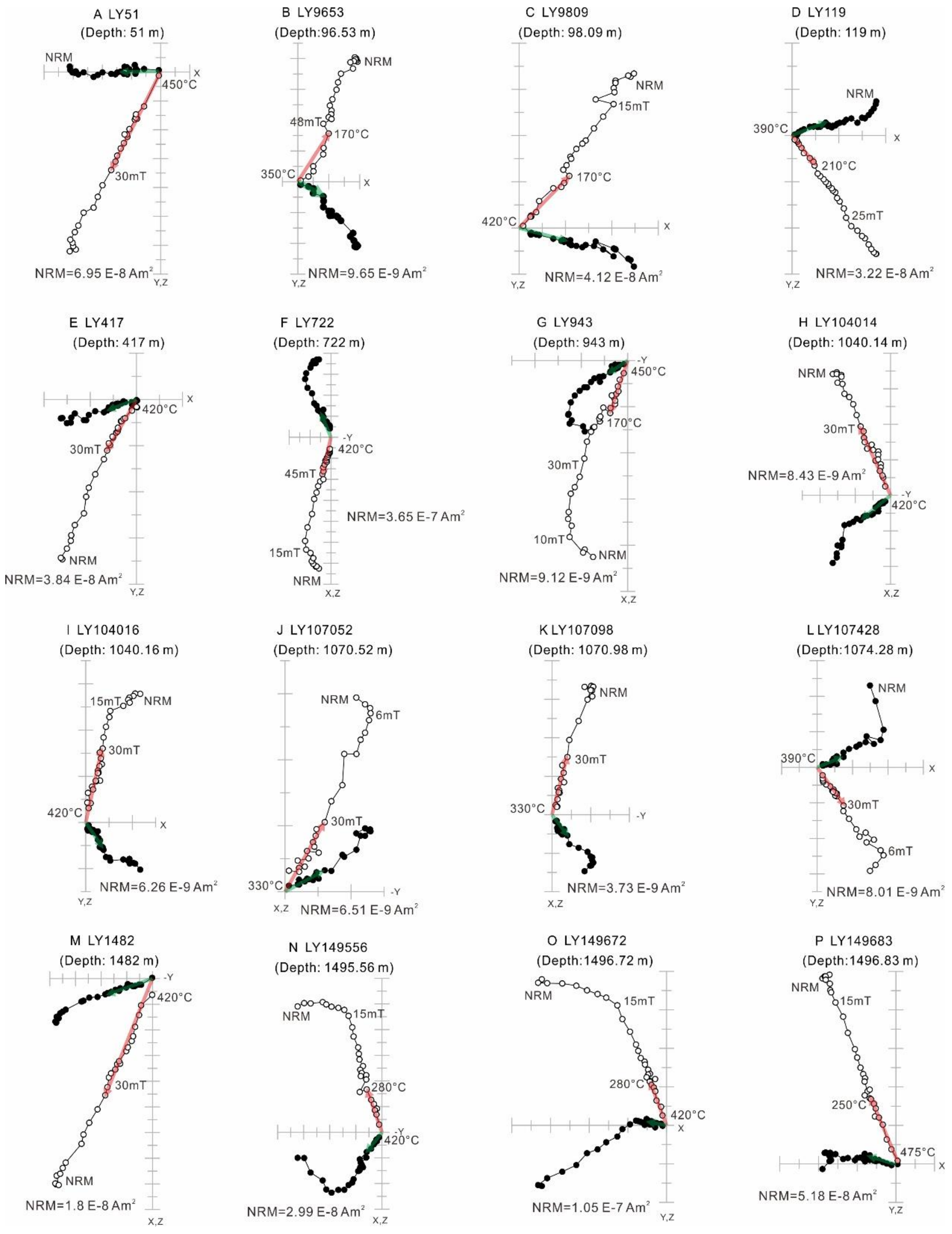

Fig. S5. Orthogonal projections of representative progressive alternating field and thermal demagnetization. The solid (open) circles refer to projections onto the horizontal (vertical) planes. The numbers refer to the temperatures in °C or alternating fields in mT. NRM is the natural remanent magnetization. Note that the magnetic declinations are arbitrary.

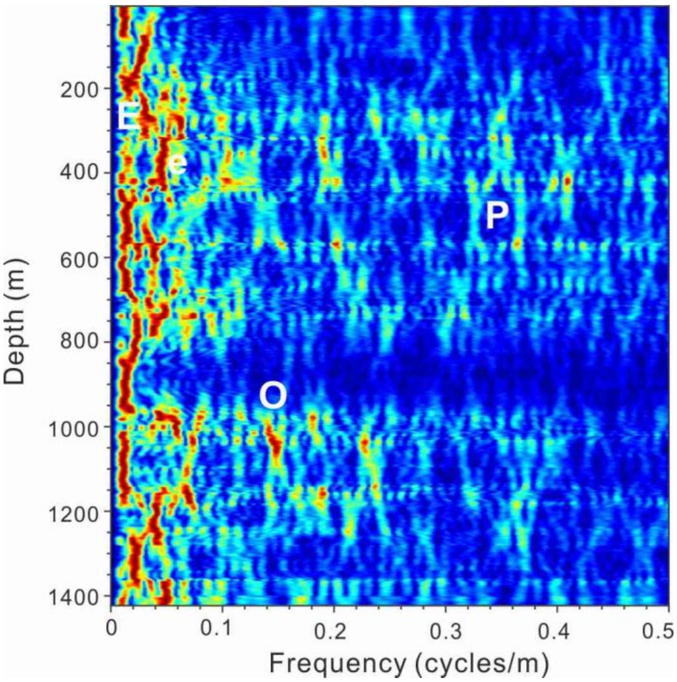

Fig. S6. Evolutionary FFT of the depth-adjusted χ_{fd}% dataset using a 120 m running window. E, long eccentricity; e, short eccentricity; O, obliquity; and P, precession.

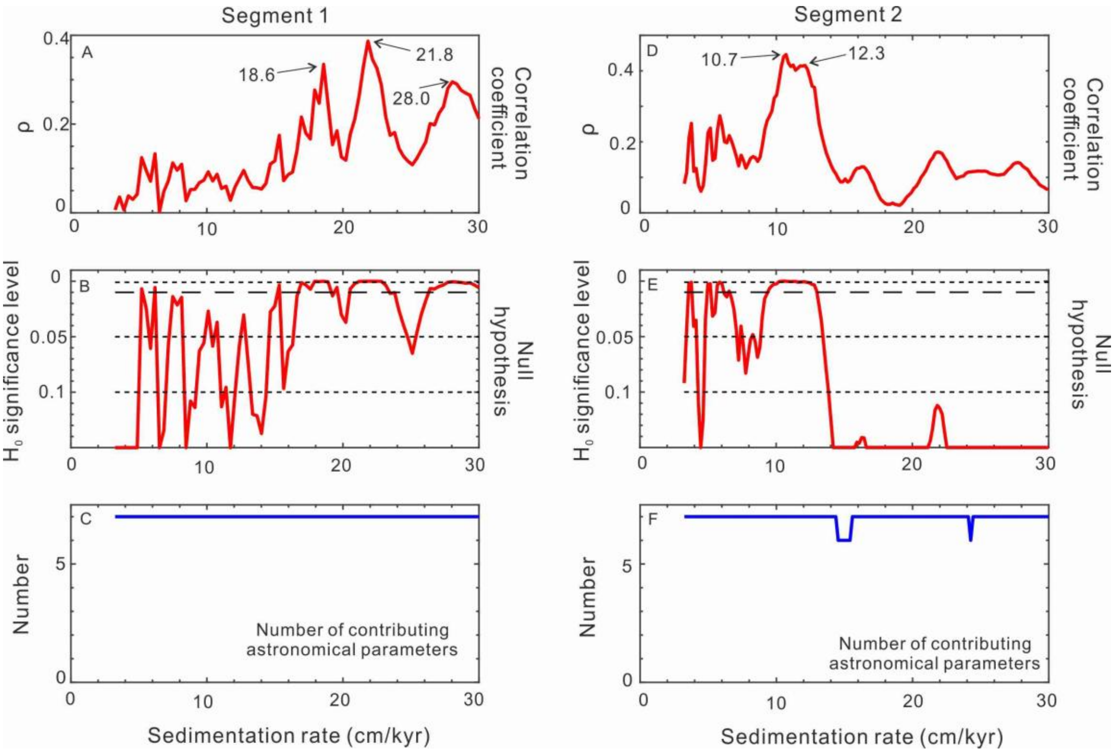

Fig. S7. Sedimentation rate estimates of the depth-adjusted χ_{fd}% series segmented by two sections. (**A, D**) Correlation coefficient (ρ) between the periodograms of the segmented χ_{fd}% series. (**B, E**) Significance level for rejecting the null hypothesis of no astronomical signals (H_0), highlighting significant sedimentation rates at 18.6 cm/kyr 21.8 cm/kyr, and 28.0 cm/kyr for Segment 1 and at 10.7 cm/kyr and 12.3 cm/kyr for Segment 2. (**C, F**) The number of contributing astronomical parameters in tested sedimentation rates.

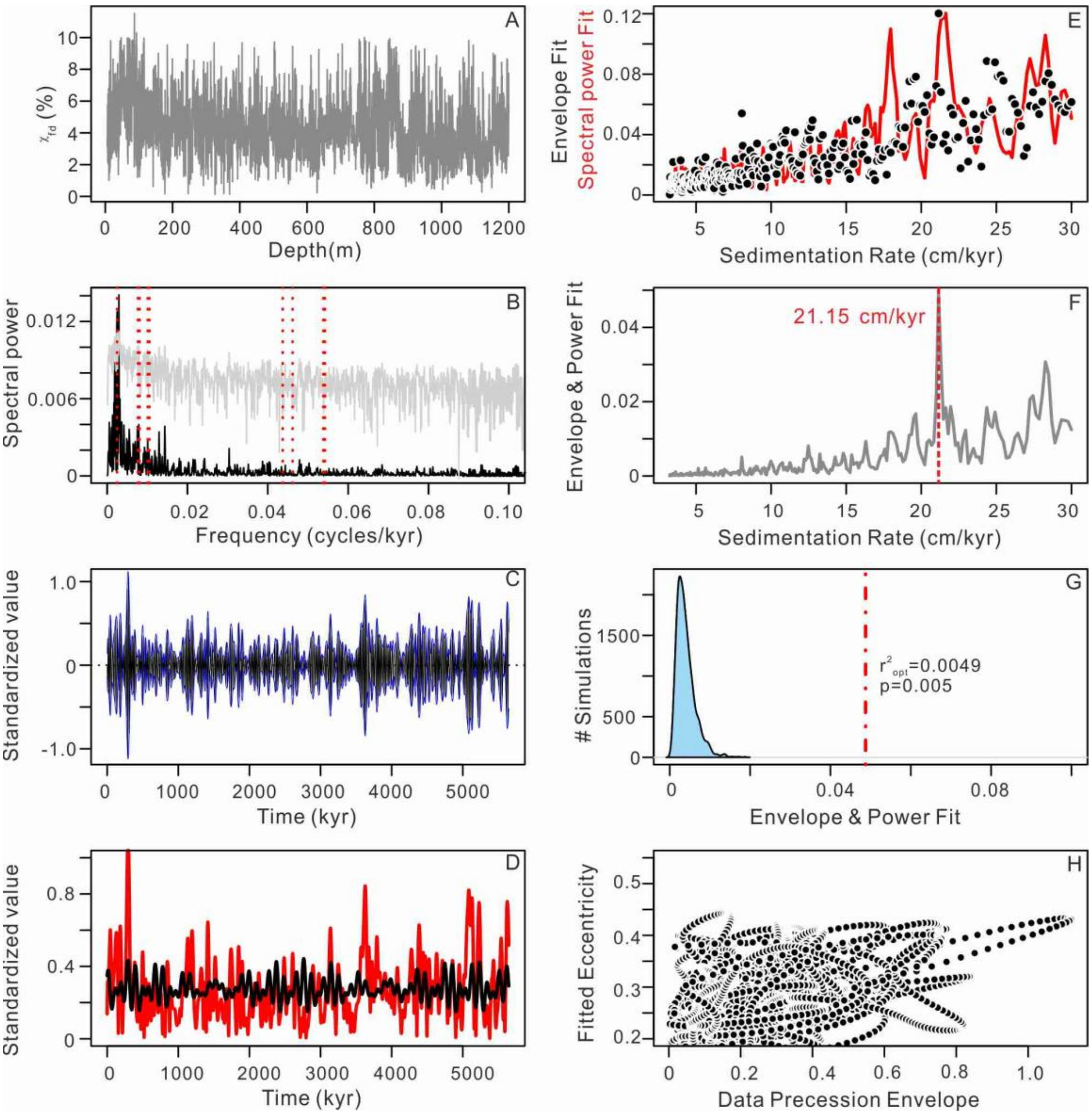

Fig. S8. TimeOpt analysis of depth-adjusted χ_{fd}% dataset for segmentation 1. (**A**) χ_{fd}% series. **(B)** Periodogram for the χ_{fd}% series, given the TimeOpt derived sedimentation rate of 21.15 cm/kyr (black line = linear spectrum; gray line = log spectrum). Dashed red lines indicate the eccentricity and precession target periods. (**C**) Comparison of the band-passed precession signal (black), and the data amplitude envelope (blue) determined via Hilbert transform. (**D**) Comparison of the data amplitude envelope (red) and the TimeOpt-reconstructed eccentricity model (black). (**E**) Squared Pearson correlation coefficient for the modulation fit ($r^2_{envelope}$; black dots) and the spectral power fit ($r^2_{spectral}$; red line) at each evaluated sedimentation rate. (**F**) Combined envelope and spectral power fit (r^2_{opt}) at each evaluated sedimentation rate. (**G**) Summary of 2000 Monte Carlo simulations with AR1 surrogates (ρ = 0.0049), used to evaluate the significance of the maximum observed r^2_{opt} of 0.005. (**H**) Cross plot of the data amplitude envelope and the TimeOpt-reconstructed eccentricity model in Figure S8D.

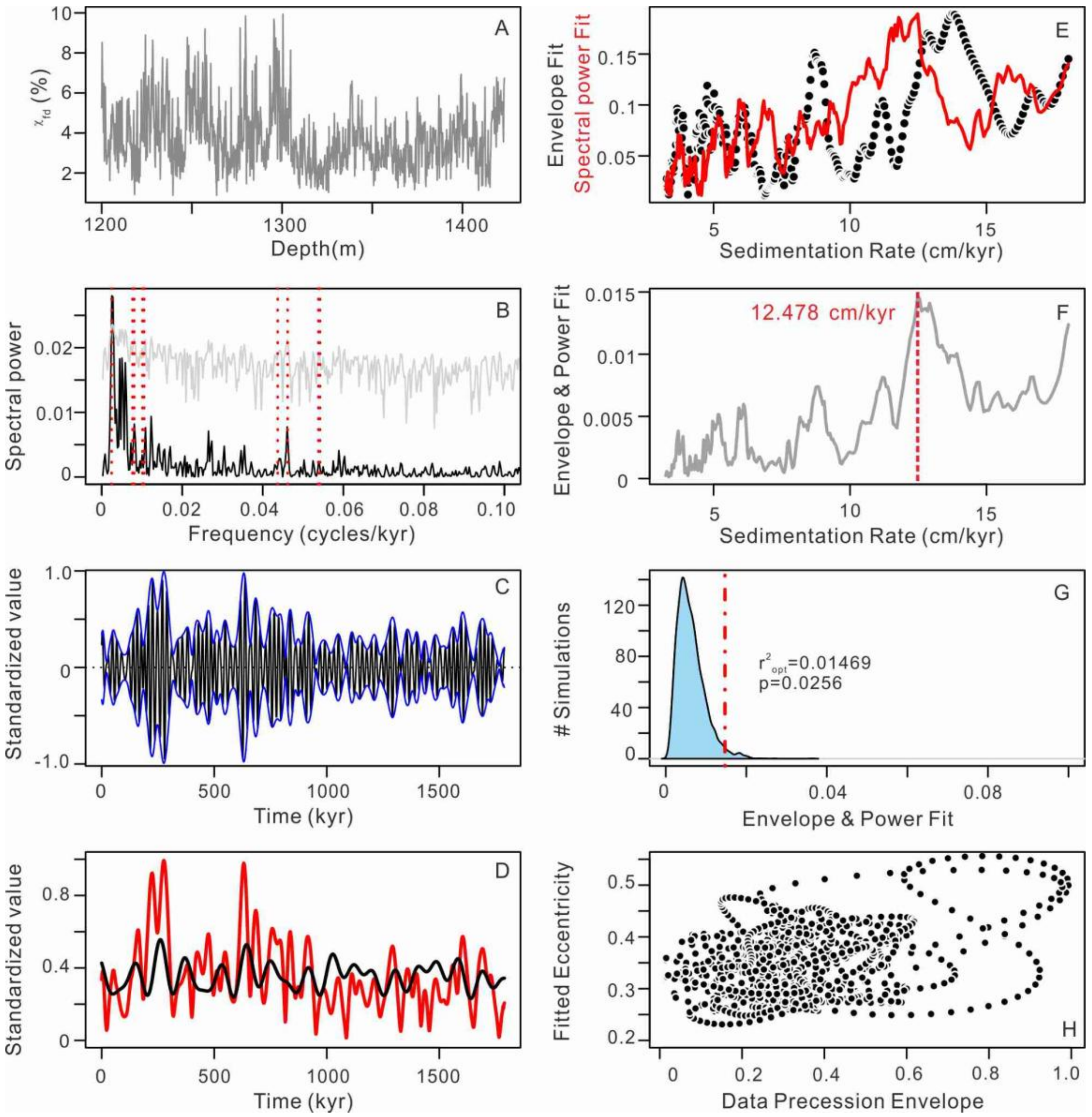

Fig. S9. TimeOpt analysis of depth-adjusted χ_{fd}% dataset for segmentation 2. (**A**) χ_{fd}% series. **(B)** Periodogram for the χ_{fd}% series, given the TimeOpt derived sedimentation rate of 12.478 cm/kyr (black line = linear spectrum; gray line = log spectrum). Dashed red lines indicate the eccentricity and precession target periods. (**C**) Comparison of the band-passed precession signal (black), and the data amplitude envelope (blue) determined via Hilbert transform. (**D**) Comparison of the data amplitude envelope (red) and the TimeOpt-reconstructed eccentricity model (black). (**E**) Squared Pearson correlation coefficient for the modulation fit ($r^2_{envelope}$; black dots) and the spectral power fit ($r^2_{spectral}$; red line) at each evaluated sedimentation rate. (**F**) Combined envelope and spectral power fit (r^2_{opt}) at each evaluated sedimentation rate. (**G**) Summary of 2000 Monte Carlo simulations with AR1 surrogates (ρ = 0.01469), used to evaluate the significance of the maximum observed r^2_{opt} of 0.0256. (**H**) Cross plot of the data amplitude envelope and the TimeOpt-reconstructed eccentricity model in Figure S9D.

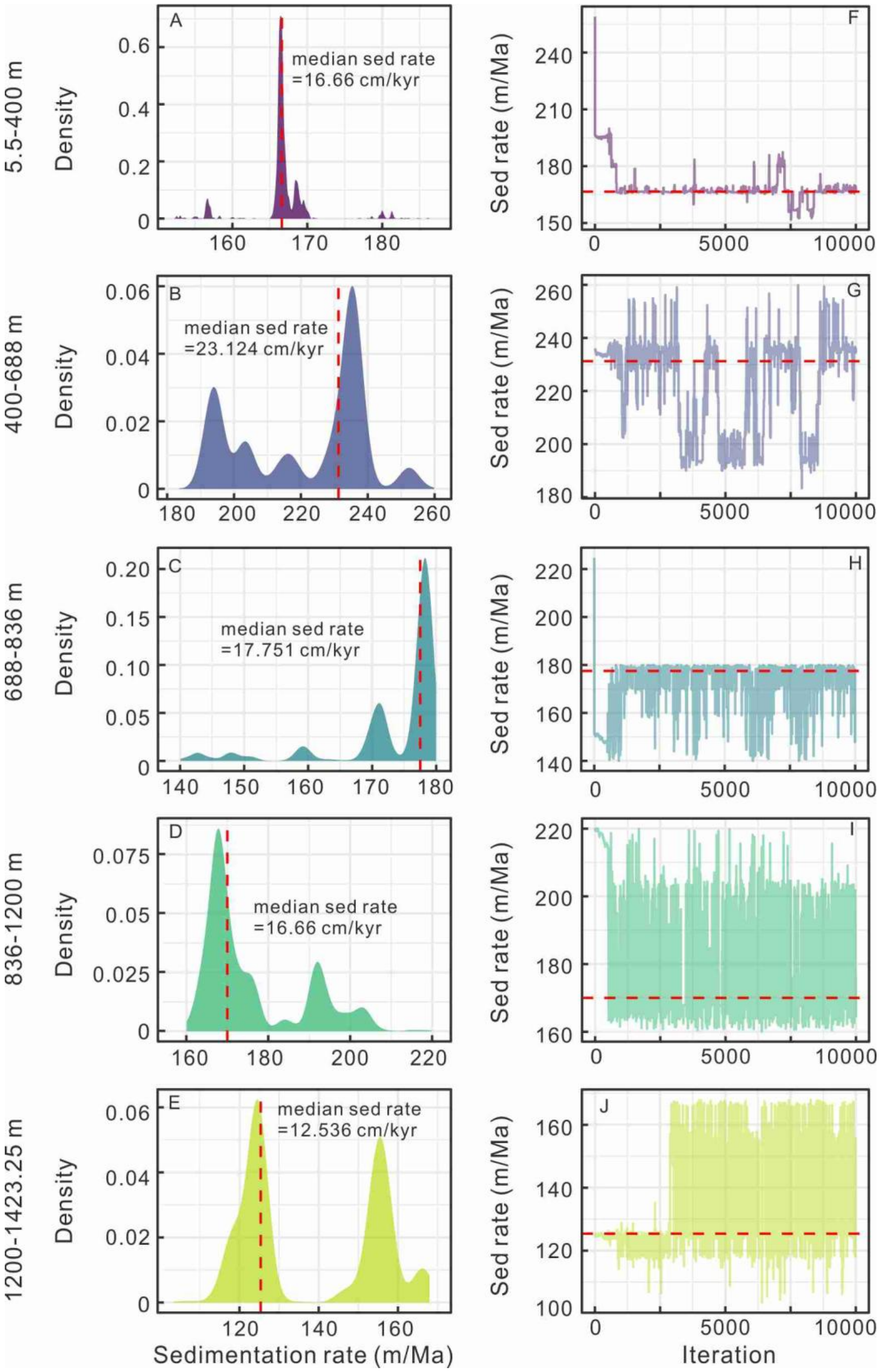

Fig. S10. AstroBayes model results for five-layer intervals. (A-E) Superimposed kernel density estimates of the posterior distribution for each model parameter. **(F-J)** Superimposed MCMC trace plots of sedimentation rate.

5. Legends for Data S1 to S4

Data S1. (separate file)

Characteristic remanent magnetization (ChRM) directions of all samples from the YSDP-4 borehole drilled from the Lower Cretaceous Jiufotang Formation in North China.

Data S2. (separate file)

The percentage of frequency-dependent magnetic susceptibility for 5889 samples from the YSDP-4 borehole drilled from the Lower Cretaceous Jiufotang Formation in North China.

Data S3. (separate file)

Orbital tuning age results for the YSDP-4 borehole.

Data S4. (separate file)

The age-depth model of the YSDP-4 borehole.

Table S1: Layer boundary positions and related minimum (Sed_min) and maximum (Sed_max) sedimentation rates

Position (m)	Sed_min (m/Myr)	Sed_max (m/Myr)
5.5	130	200
400	180	260
688	140	180
836	160	220
1200	100	170
1423.25	*NA*	*NA*

REFERENCES

1. E. Erba, C. Bottini, H. J. Weisser, C. E. Keller, Calcareous nannoplankton response to surface-water acidification around Oceanic Anoxic Event 1a. *Science* **329**, 428–432 (2010).

2. P. Menegatti, H. Weissert, High-resolution $\delta^{13}C$ stratigraphy through the early Aptian "Livello selli" of the Alpine Tethys. *Paleoceanography* **13**, 530–545 (1998).

3. E. Erba, J. E. T. Channell, M. Claps, C. Jones, R. Larson, B. Opdyke, I. P. Silva, A. Riva, G. Salvini, S. Torricelli, Integrated stratigraphy of the Cismon Apticore (southern Alps, Italy); a "reference section" for the Barremian-Aptian interval at low latitudes. *J. Foraminifer. Res.* **29**, 371–391 (1999).

4. L. M. E. Percival, L. R. Tedeschi, R. A. Creaser, C. Bottini, E. Erba, F. Girarud, H. Svensen, J. Savian, R. Trindade, R. Coccioni, F. Frontalini, L. Jovane, T. A. Mather, H. C. Jenkyns, Determining the style and provenance of magmatic activity during the early Aptian Oceanic Anoxic Event (OAE 1a). *Glob. Planet. Change* **200**, 103461 (2021).

5. Y. X. Li, T. J. Bralower, I. P. Montañez, D. A. Osleger, M. A. Arthur, D. M. Bice, T. D. Herbert, E. Erba, I. P. Silva, Toward an orbital chronology for the early Aptian Oceanic Anoxic Event (OAE1a, ~120 Ma). *Earth Planet. Sci. Lett.* **271**, 88–100 (2008).

6. K. W. Bauer, N. R. McKenzie, C. T. L. Cheung, G. Gambacorta, C. Bottini, A. R. Nordsvan, E. Erba, S. A. Crowe, A climate threshold for ocean deoxygenation during the Early Cretaceous. *Nature* **633**, 582–586 (2024).

7. B. D. A. Naafs, J. M. Castro, G. A. De Gea, M. L. Quijano, D. N. Schmidt, R. D. Pancost, Gradual and sustained carbon dioxide release during Aptian Oceanic Anoxic Event 1a. *Nat. Geosci.* **9**, 135–139 (2016).

8. C. Bottini, A. S. Cohen, E. Erba, H. C. Jenkyns, A. L. Coe, Osmium-isotope evidence for volcanism, weathering, and ocean mixing during the early Aptian OAE1a. *Geology* **40**, 583–586 (2012).

9. H. C. Jenkyns, Geochemistry of oceanic anoxic events. *Geochem. Geophys. Geosyst.* **11**, Q03004 (2010).

10. M. L. G. Tejada, K. Suzuki, J. Kuroda, R. Coccioni, J. J. Mahoney, N. Ohkouchi, T. Sakamoto, Y. Tatsumi, Ontong Java Plateau eruption as a trigger for the early Aptian oceanic anoxic event. *Geology* **37**, 855–858 (2009).

11. E. Erba, R. A. Duncan, C. Bottini, D. Tiraboschi, H. Weissert, H. C. Jenkyns, A. Malinverno, Environmental consequences of Ontong Java Plateau and Kerguelen Plateau volcanism. *Geol. Soc. Am. Spec. Paper* **511**, 271–303 (2015).

12. K. W. Bauer, R. E. Zeebe, U. G. Wortmann, Quantifying the volcanic emissions which triggered Oceanic Anoxic Event 1a and their effect on ocean acidification. *Sedimentology* **64**, 204–214 (2017).

13. P. C. Davidson, A. A. P. Koppers, T. Sano, T. Hanyu, A younger and protracted emplacement of the Ontong Java Plateau. *Science* **380**, 1185–1188 (2023).

14. Y. Li, B. S. Singer, R. Takashima, M. D. Schmitz, L. G. Podrecca, B. B. Sageman, D. Selby, T. Yamanaka, M. T. Mohr, K. Hayashi, T. Tomaru, K. Savatic, Radioisotopic chronology of Ocean Anoxic Event 1a: Framework for analysis of driving mechanisms. *Sci. Adv.* **10**, eadn8365 (2024).

15. A. Malinverno, E. Erba, T. D. Herbert, Orbital tuning as an inverse problem: Chronology of the early Aptian oceanic anoxic event 1a (Selli Level) in the Cismon APTICORE. *Paleoceanography* **25**, PA2203 (2010).

16. C. G. Leandro, J. F. Savian, M. V. L. Kochhann, D. R. Franco, R. Coccioni, F. Frontalini, S. Gardin, L. Jovane, M. Figueiredo, L. R. Tedeschi, L. Janikian, R. P. Almeida, R. I. F. Trindade, Astronomical tuning of the Aptian stage and its implications for age recalibrations and paleoclimatic events. *Nat. Commun.* **13**, 2941 (2022).

17. C. Frau, L. G. Bulot, G. Delanoy, J. A. Moreno-Bedmar, J. P. Masse, A. J. B. Tendil, C. Lanteaume, The Aptian GSSP candidate at Gorgo a Cerbara (Central Italy): An alternative

interpretation of the bio-, litho- and chemostratigraphic markers. *Newsl. Stratigr.* **51**, 311–326 (2018).

18. H. Weissert, E. Erba, Towards and Aptian GSSP. Strati2023 Book of Abstracts (11-13 July 2023; Lille, France), 114 (2023); https://strati2023.sciencesconf.org/457571.

19. H. K. H. Olierook, F. Jourdan, R. E. Merle, Age of the Barremian–Aptian boundary and onset of the Cretaceous Normal Superchron. *Earth Sci. Rev.* **197**, 102906 (2019).

20. H. Y. He, Y. X. Pan, L. Tauxe, H. F. Qin, R. X. Zhu, Toward age determination of the M0r (Barremian-Aptian boundary) of the Early Cretaceous. *Phys. Earth Planet. Inter.* **169**, 41–48 (2008).

21. Y. Zhang, J. G. Ogg, D. Minguez, M. W. Hounslow, S. Olaussen, F. M. Gradstein, S. Esmeray-Senlet, Magnetostratigraphy of U-Pb-dated boreholes in Svalbard, Norway, implies that magnetochron M0r (a proposed Barremian-Aptian boundary marker) begins at 121.2 ± 0.4 Ma. *Geology* **49**, 733–737 (2021).

22. Y. J. Li, H. F. Qin, B. R. Jicha, M. H. Huyskens, C. J. Wall, R. B. Trayler, Q. Z. Yin, M. Schmitz, Y. X. Pan, C. L. Deng, B. S. Singer, H. Y. He, R. X. Zhu, Revised onset age of magnetochron M0r: Chronostratigraphic and geologic implications. *Geology* **51**, 565–570 (2023).

23. R. Palmer, The decade of North American geology 1983 geologic time scale. *Geology* **11**, 503–504 (1983).

24. J. G. Ogg, L. A. Hinnov, C. J. Huang, “Cretaceous” in *The Geologic Time Scale 2012*, F. M. Gradstein, J. G. Ogg, M. Schmitz, G. Ogg, Eds. (Elsevier, 2012), pp. 793–853.

25. J. G. Ogg, “Geomagnetic polarity time scale” in *Geologic Time Scale 2020*, F. M. Gradsyein, J. G. Ogg, M. D. Schmitz, G. M. Ogg, Eds. (Elsevier, 2020), pp. 159–192.

26. H. Qin, W. Hao, C. Deng, P. Zhao, Z. Shen, F. Han, H. He, Y. Pan, R. Zhu, Sinistral displacement along the Tan–Lu Fault during the Cretaceous induced by Paleo-Pacific

subduction: Constraints from new paleomagnetic and U–Pb geochronological data. *J. Asian Earth Sci.* **237**, 105362 (2022).

27. B. H. Keating, C. E. Helsley, W. E. Benson, R. E. Sheridan, Paleomagnetic results from DSDP Hole 391C and the magnetostratigraphy of Cretaceous sediments from the Atlantic Ocean floor. *Init. Repts. DSDP* **44**, 523–528 (1978).

28. J. Vandenberg, C. T. Klootwijk, A. A. H. Wonders, Late Mesozoic and Cenozoic movements of the Italian Peninsula: Further paleomagnetic data from the Umbrian sequence. *Geol. Soc. Am. Bull.* **89**, 133–150 (1978).

29. J. A. Tarduno, Brief reversed polarity interval during the Cretaceous Normal Polarity Superchron. *Geology* **18**, 683–686 (1990).

30. R. X. Zhu, K. A. Hoffman, S. Nomade, P. R. Renne, R. Shi, Y. X. Pan, G. H. Shi, Geomagnetic paleointensity and direct age determination of the ISEA (M0r?) chron. *Earth Planet. Sci. Lett.* **217**, 285–295 (2004).

31. S.-c. Chang, H. Zhang, P. R. Renne, Y. Fang, High-precision $^{40}Ar/^{39}Ar$ age for the Jehol Biota. *Palaeogeogr. Palaeoclimatol. Palaeoecol.* **280**, 94–104 (2009).

32. Q. R. Meng, G. L. Wu, L. G. Fan, H. H. Wei, Tectonic evolution of early Mesozoic sedimentary basins in the North China block. *Earth Sci. Rev.* **190**, 416–438 (2019).

33. F. Y. Wu, J. H. Yang, Y. G. Xu, S. A. Wilde, R. J. Walker, Destruction of the North China Craton in the Mesozoic. *Annu. Rev. Earth Planet. Sci.* **47**, 173–195 (2019).

34. D. Y. Wang, M. X. Li, Q. Wang, L. J. Wang, Magmatic response to lithospheric thinning of the North China Craton-evidence from the Daqingshan granite. *East China Geol.* **45**, 173–186 (2024).

35. M.-D. Sun, Q. Lin, J. Ramezani, J.-S. Liu, Z.-A. Lu, H.-Q. Yang, J.-H. Bai, S.-X. Cai, J.-C. Chen, X.-Y. Chen, H. Cui, G.-C. Deng, H.-F. Gai, H.-T. Gao, J. Guo, H. Guo, L.-B. Hong, Y.-S. Hou, Y.-Y. Hua, L. Huang, K. Kong, C.-P. Li, X.-R. Liang, Z. Liu, L. Liu, B. Liu, S.-L. Liu, X.-J. Long, C. Lu, G.-M. Lu, K. Luo, S.-Y. Ma, F. Mo, Q.-L. Ouyang, X.-S. Shi, Z.-Y.

Sun, Y.-Z. Sun, H. Tang, F. Tian, H.-M. Wang, X. Wang, X. Wang, L.-W. Wang, D. Wang, Y.-X. Wei, W. Wu, C. Wu, H. Xiao, C.-M. Yang, F. Yang, B.-Y. Yin, X.-X. Yu, W.-F. Zhang, Z.-H. Zhang, R.-P. Zhao, C.-S. Zheng, Q. Zhou, Z.-Y. Zou, Z.-X. Cui, Q. Ma, L. Ma, J.-X. Zhu, J.-F. Hu, G.-J. Wei, Y.-T. Zhong, J. Shen, T. Zeng, Y.-G. Xu, Terrestrial ecosystem response to Early Cretaceous global environmental change: A calibrated, high-resolution Aptian record from Northeast China. *Earth Planet. Sci. Lett.* **653**, 119206 (2025).

36. J. M. F. Ramos, J. F. Savian, D. R. Franco, M. F. Figueiredo, C. G. Leandro, F. Frontalini, Orbital tuning of short reversed geomagnetic polarity intervals in the Cretaceous normal polarity superchron. *Geophys. Res. Lett.* **51**, e2024GL110530 (2024).

37. Z. H. Zhou, P. M. Barrett, J. Hilton, An exceptionally preserved Lower Cretaceous ecosystem. *Nature* **421**, 807–814 (2003).

38. Z. Yu, M. Wang, Y. Li, C. Deng, H. He, New geochronological constraints for the Lower Cretaceous Jiufotang Formation in Jianchang Basin, NE China, and their implications for the late Jehol Biota. *Palaeogeogr. Palaeoclimatol. Palaeoecol.* **583**, 110657 (2021).

39. X.-T. Xu, L.-Y. Shao, K. A. Eriksson, B. Pang, S. Wang, C.-X. Yang, H.-H. Hou, Terrestrial records of the early Albian Ocean Anoxic Event: Evidence from the Fuxin lacustrine basin, NE China. *Geosci. Front.* **13**, 101275 (2022).

40. R. B. Trayler, S. R. Meyers, B. B. Sageman, M. D. Schmitz, Bayesian integration of astrochronology and radioisotope geochronology. *Geochronology* **6**, 107–123 (2024).

41. K. M. Cohen, S. C. Finney, P. L. Gibbard, J. X. Fan, The ICS International Chronostratigraphic Chart. *Episodes* **36**, 199–204 (2013).

42. J. A. Tarduno, W. V. Sliter, T. J. Bralower, M. McWilliams, I. Premoli-Silva, J. G. Ogg, M-sequence reversals recorded in DSDP sediment cores from the western Mid-Pacific Mountains and Magellan Rise. *Geol. Soc. Am. Bull.* **101**, 1306–1316 (1989).

43. J. E. T. Channell, F. Cecca, E. Erba, Correlations of Hauterivian and Barremian (Early Cretaceous) stage boundaries to polarity chrons. *Earth Planet. Sci. Lett.* **134**, 125–140 (1995).

44. F. M. Gradstein, F. P. Agterberg, J. G. Ogg, J. Hardenbol, P. V. Veen, J. Thierry, Z. H. Huang, A Mesozoic time scale. *J. Geophys. Res.* **99**, 24051–24074 (1994).

45. C. J. Huang, L. Hinnov, A. G. Fischer, A. Grippo, T. Herbert, Astronomical tuning of the Aptian Stage from Italian reference sections. *Geology* **38**, 899–902 (2010).

46. Y. Wang, L. Yang, W. Shi, L. Wu, F. Wang, Qingshan sanidine (QSs): A new mesozoic $^{40}Ar/^{39}Ar$ dating standard tied to the M0r (Barremian–Aptian boundary). *Appl. Geochem.* **163**, 105932 (2024).

47. J. E. T. Channell, E. Erba, G. Muttoni, F. Tremolada, Early Cretaceous magnetic stratigraphy in the APTICORE drill core and adjacent outcrop at Cismon (Southern Alps, Italy), and correlation to the proposed Barremian-Aptian boundary stratotype. *Geol. Soc. Am. Bull.* **112**, 1430–1443 (2000).

48. A. Malinverno, J. Hildebrandt, M. Tominaga, J. E. T. Channell, M-sequence geomagnetic polarity time scale (MHTC12) that steadies global spreading rates and incorporates astrochronology constraints. *J. Geophys. Res.* **117**, B06104 (2012).

49. W. Lowrie, W. Alvarez, I. P. Silva, S. Monechi, Lower Cretaceous magnetic stratigraphy in Umbrian pelagic carbonate rocks. *Geophys. J. Int.* **60**, 263–281 (1980).

50. G. Gvirtzman, T. Weissbrod, G. Baer, G. J. Brenner, The age of the Aptian Stage and its magnetic events: New Ar-Ar ages and palaeomagnetic data from the Negev, Israel. *Cretac. Res.* **17**, 293–310 (1996).

51. J. M. Castro, P. A. Ruiz-Ortiz, G. A. de Gea, R. Aguado, I. Jarvis, H. Weissert, J. M. Molina, L. M. Nieto, R. D. Pancost, M. L. Quijiano, M. Reolid, P. W. Skelton, C. López-Rodríguez, R. Martínez-Rodríguez, High-resolution C-isotope, TOC and biostratigraphic records of OAE 1a (Aptian) from an expanded hemipelagic cored succession, western Tethys: A new

stratigraphic reference for global correlation and paleoenvironmental reconstruction. *Paleoceanogr. Paleoclimatol.* **36**, e2020PA004004 (2021).

52. S. Méhay, C. E. Keller, S. M. Bernasconi, H. Weissert, E. Erba, C. Bottini, P. A. Hochuli, A volcanic CO_2 pulse triggered the Cretaceous Oceanic Anoxic Event 1a and a biocalcification crisis. *Geology* **37**, 819–822 (2009).

53. H. Matsumoto, K. Shirai, A. Ishikawa, N. Ohkouchi, N. O. Ogawa, M. L. G. Tejada, A. Ando, J. Kuroda, K. Suzuki, Multidisciplinary evidence for synchroneity between Ontong Java Nui volcanism and early Aptian oceanic anoxic event 1a. *Sci. Adv.* **11**, eadt0204 (2025).

54. A. Ando, K. Kaiho, H. Kawahata, T. Kakegawa, Timing and magnitude of early Aptian extreme warming: Unraveling primary $\delta^{18}O$ variation in indurated pelagic carbonates at Deep Sea Drilling Project Site 463, central Pacific Ocean. *Palaeogeogr. Palaeoclimatol. Palaeoecol.* **260**, 463–476 (2008).

55. J. F. Savian, R. Trindade, L. Janikian, L. Jovane, R. P. de Almeida, R. Coccioni, F. Frontalini, M. Sideri, M. Figueiredo, The Barremian-Aptian boundary in the Poggio le Guaine core (central Italy): Evidence for magnetic polarity Chron M0r and oceanic anoxic event 1a. *Geol. Soc. Am. Spec. Paper* **524**, 57–78 (2016).

56. C. Lu, M.-Q. Lin, J. Shen, X.-K. Ji, C.-M. Yang, Z.-H. Zhang, Q. He, M.-D. Sun, Y.-G. Xu, A continental record of Early Cretaceous (Aptian) vegetation and climate change based on palynology and clay mineralogy from the North China Craton. *Palaeogeogr. Palaeoclimatol. Palaeoecol.* **662**, 112750 (2025).

57. H. F. Qin, X. Zhao, S. C. Liu, G. A. Paterson, Z. X. Jiang, S. H. Cai, J. H. Li, Q. S. Liu, R. X. Zhu, An ultra-low magnetic field thermal demagnetizer for high-precision paleomagnetism. *Earth Planets Space* **72**, 1–12 (2020).

58. J. D. A. Zijderveld, "AC demagnetization of rocks: Analysis of results" in *Methods in Paleomagnetism*, D. W. Collinson, K. M. Creer, S. K. Runcorn, Eds. (Elsevier, New York, 2013), pp. 254–286.

59. C. H. Jones, User-driven integrated software lives: “Paleomag” paleomagnetics analysis on the Macintosh. *Comput. Geosci.* **28**, 1145–1151 (2002).

60. J. L. Kirschvink, The least-squares line and plane and the analysis of palaeomagnetic data. *Geophys. J. Int.* **62**, 699–718 (1980).

61. M. S. Li, L. A. Hinnov, L. Kump, Acycle: Time-series analysis software for paleoclimate research and education. *Comput. Geosci.* **127**, 12–22 (2019).

62. S. R. Meyers, The evaluation of eccentricity-related amplitude modulation and bundling in paleoclimate data: An inverse approach for astrochronologic testing and time scale optimization. *Paleoceanography* **30**, 1625–1640 (2015).

63. W. S. Cleveland, Locally weighted regression and smoothing scatterplots. *J. Am. Stat. Assoc.* **74**, 829–836 (1979).

64. D. J. Thomson, Spectrum estimation and harmonic analysis. *Proc. IEEE* **70**, 1055–1096 (1982).

65. G. P. Weedon, K. N. Page, H. C. Jenkyns, Cyclostratigraphy, stratigraphic gaps and the duration of the Hettangian Stage (Jurassic): Insights from the Blue Lias Formation of southern Britain. *Geol. Mag.* **156**, 1469–1509 (2019).

66. Q. Jiang, M. Li, W. Yao, R. Wei, K. Ji, H. Zhang, Z. Jin, Astrochronology of the Paleocene–Eocene Thermal Maximum on the East Tasman Plateau. *Glob. Planet. Change* **252**, 104882 (2025).

67. S. R. Meyers, B. B. Sageman, Quantification of deep-time orbital forcing by average spectral misfit. *Am. J. Sci.* **307**, 773–792 (2007).

68. M. S. Li, L. R. Kump, L. A. Hinnov, M. E. Mann, Tracking variable sedimentation rates and astronomical forcing in Phanerozoic paleoclimate proxy series with evolutionary correlation coefficients and hypothesis testing. *Earth Planet. Sci. Lett.* **501**, 165–179 (2018).

69. K. P. Kodama, L. A. Hinnov, *Rock Magnetic Cyclostratigraphy* (Wiley, 2014).

70. J. Laskar, P. Robutel, F. Joutel, M. Gastineau, A. C. M. Correia, B. Levrard, A long-term numerical solution for the insolation quantities of the Earth. *Astron. Astrophys.* **428**, 261–285 (2004).

71. R. D. Müller, J. Cannon, X. Qin, R. J. Watson, M. Gurnis, S. Williams, T. Pfaffelmoser, M. Seton, S. H. J. Russell, S. Zahirovic, GPlates: Building a virtual Earth through deep time. *Geochem. Geophys. Geosyst.* **19**, 2243–2261 (2018).

72. C. R. Scotese, N. Wright, PALEOMAP Paleodigital Elevation Models (PaleoDEMS) for the Phanerozoic (PAlEOMAP Project, 2018); www.earthbyte.org/paleodem-resource-scotese-and-wright-2018/.

73. Y. Hou, H. Qin, R. N. Mitchell, Q. Li, W. Hao, M. Zhang, P. D. Ward, J. Yuan, C. Deng, R. Zhu, Completing the loop of the Late Jurassic–Early Cretaceous true polar wander event. *Nat. Commun.* **15**, 2183 (2024).

74. C. R. Pike, A. P. Roberts, K. L. Verosub, Characterizing interactions in fine magnetic particle systems using first order reversal curves. *J. Appl. Phys.* **85**, 6660–6667 (1999).

75. A. P. Roberts, C. R. Pike, K. L. Verosub, First-order reversal curve diagrams: A new tool for characterizing the magnetic properties of natural samples. *J. Geophys. Res.* **105**, 28461–28475 (2000).

76. W. Lowrie, Identification of ferromagnetic minerals in a rock by coercivity and unblocking temperature properties. *Geophys. Res. Lett.* **17**, 159–162 (1990).

77. R. J. Harrison, J. M. Feinberg, FORCinel: An improved algorithm for calculating first-order reversal curve distributions using locally weighted regression smoothing. *Geochem. Geophys. Geosyst.* **9**, Q05016 (2008).

78. D. J. Robertson, D. E. France, Discrimination of remanence-carrying minerals in mixtures, using isothermal remanent magnetisation acquisition curves. *Phys. Earth Planet. Inter.* **82**, 223–234 (1994).

79. P. P. Kruiver, M. J. Dekkers, D. Heslop, Quantification of magnetic coercivity components by the analysis of acquisition curves of isothermal remanent magnetisation. *Earth Planet. Sci. Lett.* **189**, 269–276 (2001).

80. D. Heslop, M. J. Dekkers, P. P. Kruiver, I. H. M. Van Oorschot, Analysis of isothermal remanent magnetization acquisition curves using the expectation-maximization algorithm. *Geophys. J. Int.* **148**, 58–64 (2002).

81. D. P. Maxbauer, J. M. Feinberg, D. L. Fox, MAX UnMix: A web application for unmixing magnetic coercivity distributions. *Comput. Geosci.* **95**, 140–145 (2016).

82. Z. Shen, Z. Yu, Z. Qin, D. Xi, G. Li, H. He, C. Deng, Z. Zhou, High-resolution magnetostratigraphy of the Lower Cretaceous Dabeigou Formation in the Luanping Basin, northern China. *Palaeogeogr. Palaeoclimatol. Palaeoecol.* **675**, 113021 (2025).

83. C. L. Deng, H. Y. He, Y. X. Pan, R. X. Zhu, Chronology of the terrestrial Upper Cretaceous in the Songliao Basin, northeast Asia. *Palaeogeogr. Palaeoclimatol. Palaeoecol.* **385**, 44–54 (2013).

84. C. L. Deng, R. X. Zhu, M. J. Jackson, K. L. Verosub, M. J. Singer, Variability of the temperature-dependent susceptibility of the Holocene eolian deposits in the Chinese loess plateau: A pedogenesis indicator. *Phys. Chem. Earth A* **26**, 873–878 (2001).

85. A. P. Roberts, L. Chang, C. J. Rowan, C.-S. Horng, F. Florindo, Magnetic properties of sedimentary greigite (Fe_3S_4): An update. *Rev. Geophys.* **49**, RG1002 (2011).

86. Z. Shen, Z. Yu, H. Ye, C. Deng, H. He, Magnetostratigraphy of the Upper Cretaceous Nenjiang Formation in the Songliao Basin, northeast China: Implications for age constraints on terminating the Cretaceous Normal Superchron. *Cretac. Res.* **135**, 105213 (2022).

87. L. Tauxe, *Essentials of Paleomagnetism* (University of California Press, 2010).

88. A. P. Roberts, R. Weaver, Multiple mechanisms of remagnetization involving sedimentary greigite (Fe3S4). *Earth Planet. Sci. Lett.* **231**, 263–277 (2005).

89. L. Chang, A. P. Roberts, Y. Tang, B. D. Rainford, A. R. Muxworthy, Q. Chen, Fundamental magnetic parameters from pure synthetic greigite (Fe_3S_4). *J. Geophys. Res.* **113**, B06104 (2008).

90. H. He, C. Deng, P. Wang, Y. Pan, R. Zhu, Toward age determination of the termination of the Cretaceous Normal Superchron. *Geochem. Geophys. Geosyst.* **13**, Q02002 (2012).

91. A. R. Muxworthy, D. J. Dunlop, First-order reversal curve (FORC) diagrams for pseudo-single-domain magnetites at high temperature. *Earth Planet. Sci. Lett.* **203**, 369–382 (2002).

92. A. P. Roberts, Q. Liu, C. J. Rowan, L. Chang, C. Carvallo, J. Torrent, C.-S. Horng, Characterization of hematite (α-Fe_2O_3), goethite (α-FeOOH), greigite (Fe_3S_4), and pyrrhotite (Fe_7S_8) using first-order reversal curve diagrams. *J. Geophys. Res.* **111**, B12S35 (2006).

93. J. A. Dearing, K. L. Hay, S. M. J. Bahan, A. S. Huddleston, E. M. H. Wellington, P. J. Loveland, Magnetic susceptibility of soil: An evaluation of conflicting theories using a national data set. *Geophys. J. Int.* **127**, 728–734 (1996).

94. B. A. Maher, R. M. Taylor, Formation of ultrafine-grained magnetite in soils. *Nature* **336**, 368–370 (1988).

95. L. P. Zhou, F. Oldfield, A. G. Wintle, S. G. Robinson, J. T. Wang, Partly pedogenic origin of magnetic variations in Chinese loess. *Nature* **346**, 737–739 (1990).

96. X. M. Liu, T. S. Liu, P. Hesse, D. S. Xia, J. Chalachula, G. Wang, Two pedogenic models for paleoclimatic records of magnetic susceptibility from Chinese and Siberian loess. *Sci. China Ser. D Earth Sci.* **51**, 284–293 (2008).

97. C. Deng, N. J. Vidic, K. L. Verosub, M. J. Singer, Q. Liu, J. Shaw, R. Zhu, Mineral magnetic variation of the Jiaodao Chinese loess/paleosol sequence and its bearing on long-term climatic variability. *J. Geophys. Res.* **110**, B03103 (2005).

98. T. Zhang, Y. Li, T. Fan, A.-C. Da Silva, J. Shi, Q. Gao, M. Kuang, W. Liu, Z. Gao, M. Li, Orbitally-paced climate change in the early Cambrian and its implications for the history of the Solar System. *Earth Planet. Sci. Lett.* **583**, 117420 (2022).

99. H. Zhang, Y. Chen, J. G. Ogg, Z. Sun, P. B. Wignall, M. Wang, H. X. Zhang, X. Y. Zhang, Y. Zhang, K. K. Huang, H. L. Lu, B. C. Huang, M. S. Li, Astronomically calibrated integrated stratigraphy of the Induan Stage (Early Triassic) and significance for the Permian–Triassic mass extinction and aftermath. *Earth Planet. Sci. Lett.* **669**, 119563 (2025).
